# Large-particle aerosol exposure to the Bangladesh or Malaysia strain of Nipah virus results in markedly divergent disease presentation in African Green Monkeys

**DOI:** 10.1371/journal.ppat.1013835

**Published:** 2025-12-29

**Authors:** Yu Cong, Jeremy Bearss, Venkatesh Mani, Matthew Lackemeyer, Bapi Pahar, Louis M. Huzella, Erin Kollins, Steve Mazur, Saurabh Dixit, Sanae Lembirik, David Drawbaugh, Philip J. Sayre, Joseph Laux, Jeffrey Solomon, Dima A. Hammoud, Ji Hyun Lee, Claudia Calcagno, Russ Byrum, Marisa St. Claire, Jiro Wada, Vincent Munster, Michael R. Holbrook

**Affiliations:** 1 Integrated Research Facility at Fort Detrick, National Institute of Allergy and Infectious Diseases, National Institutes of Health, Fort Detrick, Frederick, Maryland, United States of America; 2 Clinical Monitoring Research Program Directorate, Frederick National Laboratory for Cancer Research, Frederick, Maryland, United States of America; 3 Clinical Center, Center for Infectious Disease Imaging, National Institutes of Health, Bethesda, Maryland, United States of America; 4 Clinical Center, Radiology and Imaging Sciences, National Institutes of Health, Bethesda, Maryland, United States of America; 5 Virus Ecology Unit, Laboratory of Virology, National Institute of Allergy and Infectious Diseases, National Institutes of Health, Hamilton, Montana, United States of America; University of Texas Medical Branch / Galveston National Laboratory, UNITED STATES OF AMERICA

## Abstract

Nipah virus (NiV), a highly pathogenic zoonotic paramyxovirus, causes severe respiratory and neurological disease in humans, with a case-fatality rate around 60%. Descriptions of cases in the clinical setting suggest that the two primary lineages of NiV cause disease with different presentations and outcomes. To define strain-specific differences in disease progression and host responses, African green monkeys were exposed to either the Malaysia (NiV-M) or Bangladesh (NiV-B) strain using a large-particle aerosol exposure. NiV-M infection resulted in a fatality rate of 27%, while NiV-B infection led to a 75% fatality rate characterized by rapid respiratory decline and systemic viral dissemination. Among survivors, NiV-M–infected animals mounted robust immunoglobulin M, immunoglobulin G, and neutralizing antibody responses, whereas NiV-B survivors exhibited weaker and delayed humoral responses. Non-survivors of both strains showed elevated pro-inflammatory cytokines, thrombocytopenia, and multi-organ dysfunction. Imaging showed that NiV-M infection was associated with neuroinflammation and systemic vasculitis, while NiV-B infection caused progressive pulmonary pathology. Histopathological analysis confirmed widespread vasculitis and encephalitis in animals with NiV-M infection and diffuse pulmonary hemorrhage and fibrin thrombi, consistent with vascular injury and coagulopathy, in animals with NiV-B infection. Cytokine profiling and flow cytometry showed a more intense and dysregulated immune response to NiV-B infection. Fatal outcomes in both groups were associated with thrombocytopenia, elevated pro-inflammatory cytokines, and multi-organ dysfunction. This study highlights fundamental differences in virulence, immune evasion, and pathogenesis between NiV strains and underscores the value of the African green monkey aerosol model for evaluating medical countermeasures under conditions that closely mimic natural human exposure.

## Author summary

Nipah virus (NiV) is a highly lethal zoonotic pathogen that causes respiratory and neurological disease in humans. There are no approved treatments or vaccines and understanding how different strains of NiV cause disease is critical for developing effective medical countermeasures. In this study, we used African green monkeys, a well-established nonhuman primate model, to compare the progression of disease caused by two NiV strains: Malaysia (NiV-M) and Bangladesh (NiV-B). We used a large-particle aerosol exposure method to mimic natural infection, which occurs via inhalation of droplets. NiV-B caused rapid respiratory failure, minimal antibody responses, and high fatality. In contrast, NiV-M led to a slower disease course, and some animals survived with strong antibody responses and signs of neurological involvement. Blood tests, imaging, and tissue analyses revealed clear differences in the immune response to each strain: NiV-B triggered an early, intense inflammatory response, while NiV-M evaded the immune system longer before causing damage. These findings provide insight and highlight differences in disease severity, immune response, and clinical outcomes of the two NiV strains. Our results reinforce the value of the African green monkey model for testing future NiV vaccines and treatments for infection.

## Introduction

In 1998, a novel paramyxovirus was identified in Malaysia during an outbreak of neurological disease in people and respiratory disease in pigs [1,2]. This virus, Nipah virus (NiV), was found to be closely related to Hendra virus (HeV), which had been identified a few years prior in Australian horses with evidence of neurological disease [3,4]. While NiV has not been seen in Malaysia since the original outbreak, the virus has appeared annually in Bangladesh and/or India since 2001 [5]. Bats within the *Pteropus* genus have been identified as the likely primary reservoir for both NiV and HeV [6,7]. Detection of complete NiV genomes has demonstrated continued circulation in bats collection in Malaysia, India, Bangladesh, Thailand, and Cambodia [8].

NiV transmission to the human population in Malaysia occurred primarily with pigs as the intermediary host of the virus [9]. Bats roosted over piggeries, into which they would urinate, defecate, and drop partially eaten fruit. The pigs developed severe respiratory disease with significant nasal discharge, which facilitated transmission to individuals working near the pigs [9]. It is thought that, in Bangladesh, NiV infection in the human population occurs through the consumption of date palm sap [10], although human-to-human transmission has also been documented [11]. Transmission of the virus among humans is likely through inhalation of droplets rather than through the alimentary tract. In India, the mechanism of human infection is less clear because date palm sap is not the delicacy that it is in Bangladesh. Direct contact with infected bats seems to be the most probable scenario for transmission to humans [12,13].

NiV genetic sequences segregate into two distinct lineages (I and II) that emerged in ~1937, with minor genotypes diverging in the 1970s [14,15]. The Malaysia strain (NiV-M) and Bangladesh strain (NiV-B) have been shown to induce differential transcriptomic profiles in infected pulmonary epithelial cells [16,17]. Laboratory studies in African green monkeys (AGMs) and Syrian Golden hamsters suggest that there is a difference in the pathogenicity of the NiV-M and NiV-B [18,19]. Some of these data correlate with anecdotal evidence suggesting that people infected with NiV-M had a higher propensity to develop neurological disease, while those infected with NiV-B developed both respiratory and neurological disease. The high case-fatality rate (≈60%) and potential for human-to-human transmission highlight the urgent need for effective antiviral strategies to combat this life-threatening pathogen. Currently, there is limited understanding of the mechanisms underlying NiV infection, and no approved therapeutic options or vaccines are available.

Previous work by our group using intratracheal (IT) and small-particle (≈3 μm) aerosol exposure of AGMs to NiV-M led to a rapidly progressing severe respiratory disease with marked evidence of hemorrhage [20]. Further work with intermediate particle (≈7 μm), NiV-M aerosol exposure led to an extended disease, with one survivor that showed evidence of microinfarctions and encephalitis when evaluated by magnetic resonance imaging (MRI) [21]. Studies by other groups have shown clear variability in the disease caused by NiV-M and NiV-B in the AGM model using inoculation via a combination of IT and intranasal (IN) routes [19].

The overall scope of this project was to evaluate and describe how two epidemiologically distinct Nipah strains behave under the same large-particle aerosol exposure conditions in a highly susceptible NHP host in an effort to recapitulate a probable human exposure route. In the work described here, we evaluated low-dose (≈500 PFU), large-particle (≈12 μm) aerosol exposure of AGMs to either NiV-M or NiV-B. We found markedly different disease presentation, with higher survival, a longer disease course, and evidence of neurological disease, in animals exposed to NiV-M; however, most animals exposed to NiV-B developed a very rapidly progressing respiratory disease, with a much lower survival rate. In the NiV-M exposure group, despite the succumbed animals, most of the surviving animals got infected, but a few animals did not become infected, as evidenced by a lack of clinical signs of disease and no seroconversion. In the NiV-B exposure group, animals either succumbed after a rapidly progressing respiratory disease and were found deceased at early morning observations, or met endpoint criteria and were euthanized, or survived and showed no evidence of infection. In addition, consistent with previous work, imaging showed distinct tissue tropism: NiV-M infection was associated with neuroinflammation and vasculitis, while NiV-B infection led to progressive pulmonary pathology [22].

These data clearly demonstrate a significant difference in the disease caused by NiV-M and NiV-B in the AGM model. The data generated here suggest that large-particle aerosol exposure to NiV more accurately reflects the nature of transmission to humans than any other exposure routes reported. This animal disease model and route of exposure are valuable for examining mechanisms of viral pathogenesis and developing virus- or host-specific medical countermeasures for treating NiV infection.

## Results

**Fig 1:**
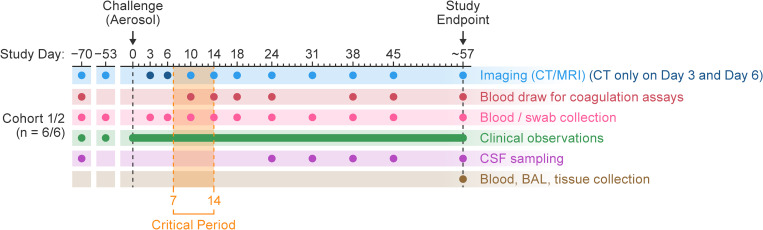
Study design for comparative pathogenesis of NiV-M and NiV-B in AGMs. Adult AGMs were exposed to either NiV-M or NiV-B via large-particle aerosol (≈500 PFU) to assess strain-specific disease progression. Two groups of 12 animals (two cohorts NiV-M; and two cohorts for NiV-B) were used; Two later cohorts repeated the initial exposures to both strains to increase statistical power. Clinical monitoring and scheduled sampling (blood, swabs, CSF) were conducted at baseline and multiple time points post-exposure through the end of the study. Whole-body CT and brain MRI were performed longitudinally, with early-phase MRI omitted in later cohorts due to anesthesia-related complications. Imaging and clinical data were aligned by study week after the acute phase for consistency across cohorts. BAL, bronchoalveolar lavage; CSF, cerebral spinal fluid; CT, computed tomography; AGMs, African green monkeys; MRI, magnetic resonance imaging; NiV, Nipah virus; NiV-B, Nipah virus Bangladesh strain; NiV-M, Nipah virus Malaysia strain.

This study was designed and carried out in four cohorts. The first two, NiV-M cohort 1 (*n* = 6) and NiV-B cohort 1 (*n* = 6) exposure, were conducted about 4 years prior to the second two cohorts (*n* = 6 each) due to an interruption of work caused by the COVID pandemic. The experimental plan was almost the same for all four cohorts (Fig 1), however, adjustments in time points were made for NiV-M cohort 2 and both cohorts of NiV-B because animals’ intolerance for frequent and prolonged anesthesia sessions was found during the conduct of work with NiV-M cohort 1.

### Clinical disease

**Fig 2:**
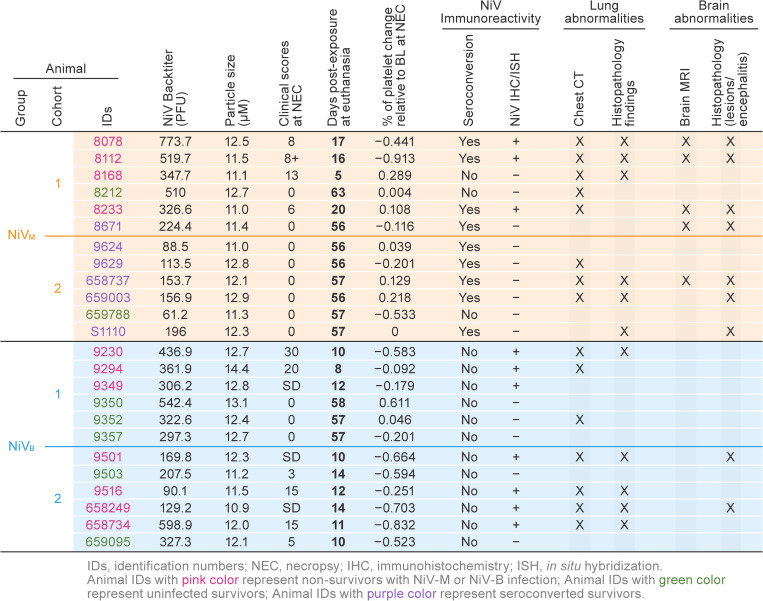
Comparative summary of clinical, virological, and imaging findings in cynomolgus and rhesus macaques following NiV-M or NiV-B exposure. Summary data for all animals included in this study including dose given, particle size at exposure, clinical scores and day post exposure at euthanasia, comparative change in platelets and various clinical assessments.

Animals were exposed to a target dose of 500 PFU NiV (NiV-M: achieved dose of 61–774 PFU, mean = 289 PFU; NiV-B achieved dose of 90–599 PFU, mean = 316 PFU), with an average particle size of 12.1 µm across all four cohorts (Fig 2).

**Fig 3:**
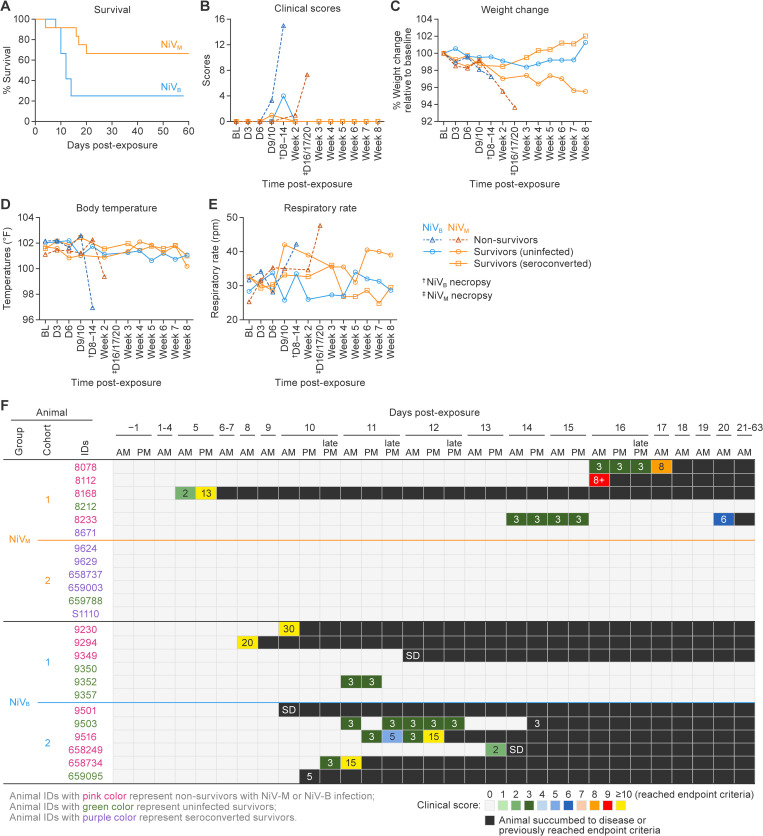
Clinical progression in AGMs following NiV-M or NiV-B aerosol exposure. **(A)** Kaplan-Meier curves show survival of AGMs exposed to NiV-M (*n* = 12) or NiV-B (*n* = 12) via large-particle aerosol. NiV-B led to a shorter disease course (Median survival time = 12 d) compared to NiV-M (Median survival time = 17 d). **(B)** Animals were grouped as uninfected survivors (circles), non-survivors (triangles), or seroconverted survivors (squares) based on virological and clinical outcomes. Clinical scores indicate rapid disease progression after onset. Neurologic signs were seen only in animals with NiV-M infection. **(C)** Percent weight changes relative to baseline. **(D)** Temperature profiles. Animals with NiV-M infection showed transient fevers (10–14 d) and hypothermia at euthanasia. NiV-B animals showed no fever but had a sharper terminal temperature drop. **(E)** Respiratory rates increased significantly in non-survivors exposed to either strain, consistent with pulmonary pathology. Rates remained unchanged in survivors and uninfected animals. Data (except survival) are presented as group means. **(F)** Longitudinal clinical scores and outcome timeline of cynomolgus and rhesus macaques following NiV-M or NiV-B infection.

In the NiV-M-exposed cohorts, 67% of the NiV-M-exposed animals (eight out of 12) survived, with two survivors uninfected as evident by serology (Figs 2, 3A, 3B, and 3F). A total of four animals (33%) met endpoint criteria and were euthanized at 5, 16, 17, and 20 d (average survival time [AST] = 17.7 d). Specifically, at 16–20 d, three of these animals developed a similar clinical syndrome, which culminated in neurologic signs consisting of intention tremors, eyelid twitches, and generalized shaking along with progressive weakness and non-responsiveness. The fourth animal (8168) developed an acute respiratory syndrome, consisting of fever, rapid respiration, and dehydration; this animal was euthanized at 5 d and excluded from post-life analysis. Subsequently, the survival rate was adjusted to 73% (eight survivors out of 11).

**Fig 4:**
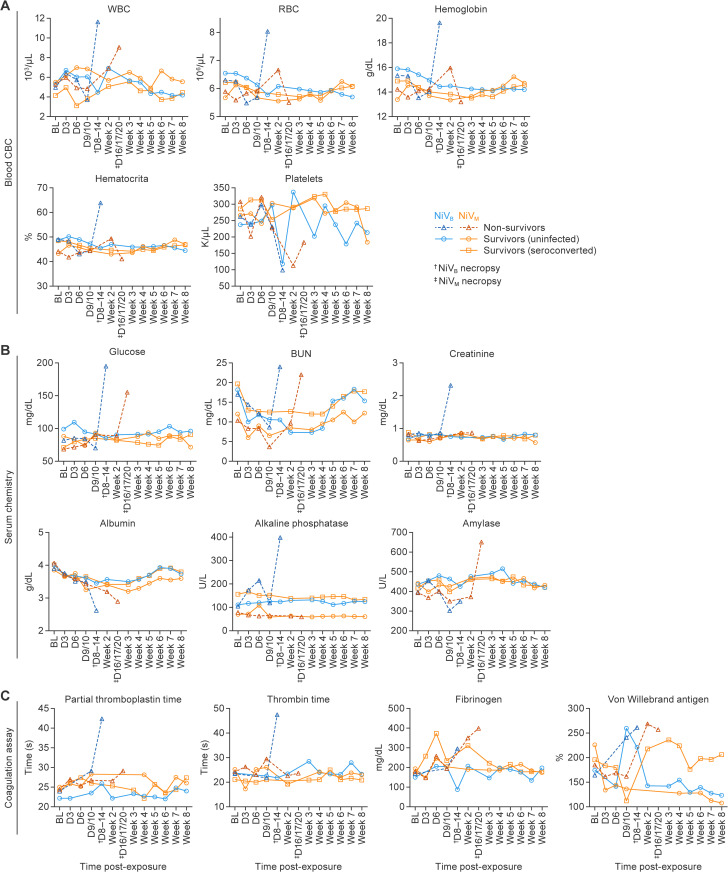
Hematologic, biochemical, and coagulation changes in AGMs following NiV-M and NiV-B exposure. Animals were grouped as uninfected survivors (circles), non-survivors (triangles), or seroconverted survivors (squares) based on virological and clinical outcomes. **(A)** Time course of hematologic parameters (white blood cells, red blood cells, hemoglobin, hematocrit, and platelets) in NiV-M, NiV-B, and uninfected animals. Non-survivors exposed to NiV-M and NiV-B showed significant thrombocytopenia at terminal time points compared to their own baselines or uninfected survivors. Uninfected animals remained stable. **(B)** Longitudinal serum chemistry profiles, including glucose, BUN, creatinine, ALP, amylase, and albumin. NiV-B-exposed animals with fatal outcomes exhibited marked elevations in BUN, creatinine, and liver enzymes consistent with multi-organ dysfunction. Survivors and uninfected animals showed minimal changes. **(C)** Coagulation parameters: Partial Thromboplastin Time (PTT), thrombin time (TT), fibrinogen, and VWF antigen. Animals with NiV-B infection exhibited prolonged PTT/TT and elevated VWF antigen levels compared to those with NiV-M infection and uninfected groups, indicating greater endothelial dysfunction and coagulopathy. Data are presented as group means.

**Fig 5:**
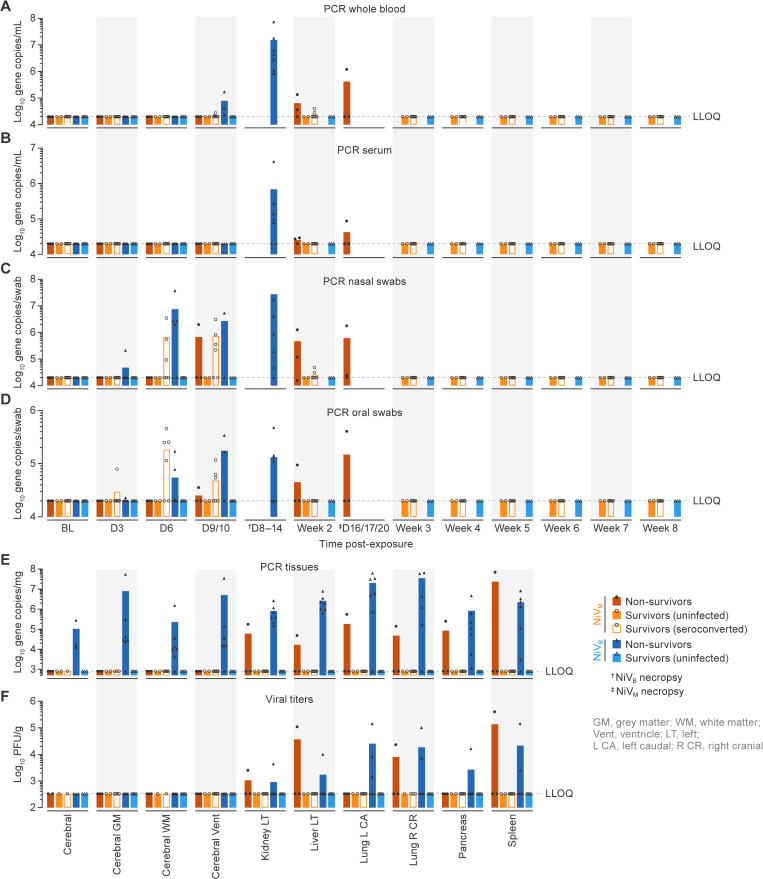
Viral RNA load and tissue dissemination in AGMs following NiV-M and NiV-B exposure. Animals were grouped as uninfected survivors (circles), non-survivors (triangles), or seroconverted survivors (squares) based on virological and clinical outcomes. Quantification of viral RNA (vRNA) in whole blood **(A)**, serum **(B)**, nasal swabs **(C)**, and oral swabs **(D)** over time. Survivors had undetectable vRNA in all sample types. **(E)** Detection of vRNA by RT-qPCR and **(F)** viable virus in tissues by plaque assay. NiV viral RNA and live virus were detected only in non-survivors, with widespread distribution across multiple organs. All survivors were negative by both assays, consistent with complete viral clearance at necropsy. Data are presented as group means with values of each animal.

**Fig 6:**
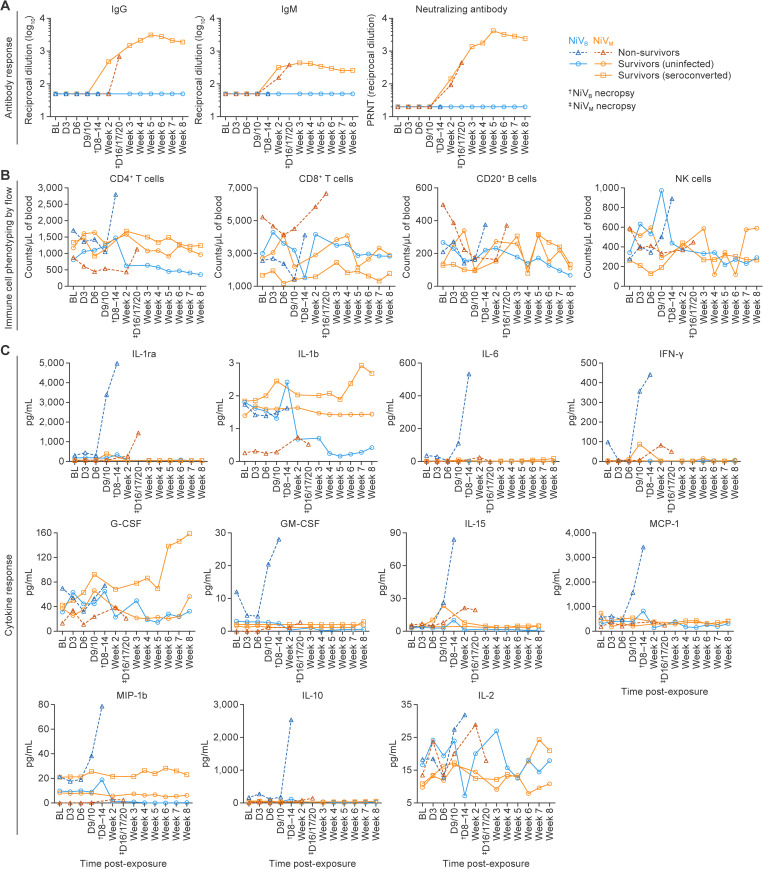
Host immune responses to NiV-M and NiV-B infection in AGMs. Animals were grouped as uninfected survivors (circles), non-survivors (triangles), or seroconverted survivors (squares) based on virological and clinical outcomes. **(A)** Serological responses to NiV-M and NiV-B exposure. Animals with NiV-M infection developed IgM, IgG, and neutralizing antibodies; survivors maintained robust titers through study end. Two survivors lacked detectable antibodies, indicating no infection. In NiV-M non-survivors, IgM responses were present, but IgG and neutralizing responses were delayed or insufficient. In contrast, no antibodies were detected in NiV-B-exposed animals, indicating disease progression outpaced humoral immunity. Seroconversion rates were significantly higher in NiV-M compared to NiV-B. **(B)** Longitudinal immune cell dynamics measured by flow cytometry. NiV-M non-survivors showed early lymphocyte depletion (CD4 + , CD8 + , B, and NK cells) with terminal-phase rebound; survivors showed similar trends, while uninfected animals remained stable. In NiV-B non-survivors, CD4 + T cells increased at terminal time points, with suppressed CD8 + T cell and B-cell populations earlier in the disease. No significant changes were observed in uninfected animals. Due to the small sample size and inter-animal variation, statistical comparisons were not performed. **(C)** Plasma cytokine profiles. Non-survivors exposed to either NiV strain showed sharp increases in IFN-γ, IL-15, and IL-1RA. NiV-B non-survivors also had elevated IL-6, IL-10, G-CSF, MCP-1 and MIP-1β. Survivors of NiV-M infection showed transient cytokine elevations returning to baseline by 10–14 d and sustained increases in G-CSF. Data are presented as group means.

**Fig 7:**
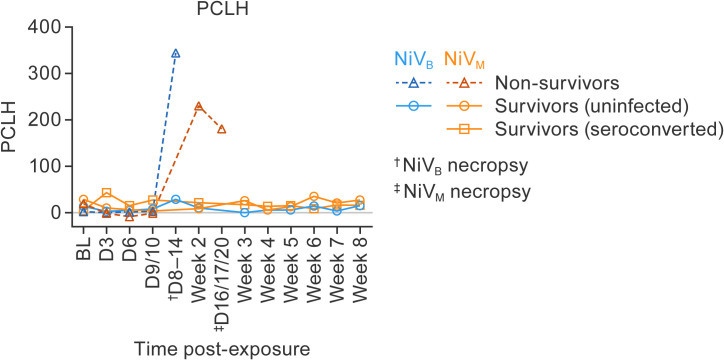
Quantitative CT analysis of lung pathology in AGMs following NiV exposure. Percent change in lung hyperdensity (PCLH) was quantified over time using serial chest CT imaging to assess pulmonary disease progression in AGMs exposed to NiV-M or NiV-B. Animals were grouped as uninfected survivors (circles), non-survivors (triangles), or seroconverted survivors (squares) based on virological and clinical outcomes. NiV-B non-survivors (n = 7) showed a significant increase in PCLH, with peak values reaching over 350%, compared to NiV-M non-survivors (n = 3), which showed peak PCLH around 200%. In contrast, both uninfected animals (NiV-M: n = 2; NiV-B: n = 5) and seroconverted survivors (NiV-M: n = 6) showed minimal changes in PCLH throughout the study period. A significant difference in peak PCLH was observed between NiV-B non-survivors and all other groups. These findings indicate that severe lung hyperdensity changes detected by CT imaging are associated with fatal disease progression in NiV-B–exposed animals. Data are presented as group means.

**Fig 8:**
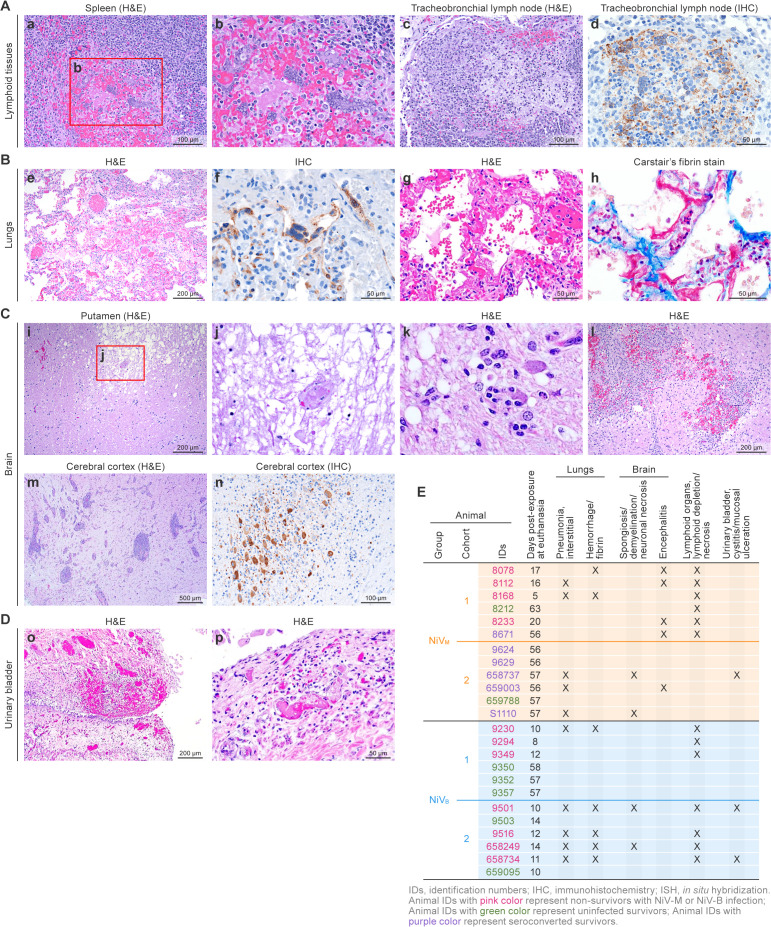
Histopathologic lesions and NiV antigen distribution in multiple tissues of AGMs with NiV infection. **(A: a–d)** Lymphoid tissues (animal 09230): (a, b) Spleen: white pulp germinal center depletion, hemorrhage, and syncytial cell formation (hematoxylin and eosin stain (HE), 200X [a] and 400X [b]). (c) Tracheobronchial lymph node: follicular lymphoid depletion with necrosis and hemorrhage (HE, 200X). (d) NiV antigen in affected follicles (IHC, 400X). **(B: e–h)** Lung tissues (animals 08112 and 9501): (e) Necrotizing interstitial pneumonia with hemorrhage and edema (HE, 100X). (f) NiV antigen within pneumocytes and syncytial cells (IHC, 400X). (g) Interstitial pneumonia with fibrin and hyaline membrane formation (HE, 200X). (h) Hyaline membranes highlighted using Carstair’s fibrin stain (400X). **(C: i–n)** Brain tissues (animals 08078, 08233 and 8671): (i, j) Putamen: encephalomalacia and vessel occlusion by fibrin thrombus (HE, 100X and 400X). (k) Neuronophagia and neuropil vacuolation. (l) Brainstem from 8671 showing moderate necrotizing, lymphohistiocytic multifocal encephalitis with hemorrhage. (m) Cerebral cortex: Mild-to-moderate non-suppurative encephalitis and meningitis (HE, 100X). Lesions were multifocal and were the most severe in and surrounding the olfactory tubercle. (n) NiV antigen in neurons and glial cells (IHC, 200X). **(D: o–p)** Urinary bladder (animal 09501): (o) Submucosal hemorrhage, edema, and lymphocytic inflammation (HE, 40X). (p) Vessel occlusion with fibrin thrombus (HE, 100X).

In the NiV-B-exposed cohorts, clinical scores demonstrated that infection led to a rapidly progressing disease, with animals reaching euthanasia criteria within a few days of disease onset. Indeed, 75% of animals (nine out of 12) either succumbed to the disease or were euthanized after meeting endpoint criteria at 8–14 d. Specifically, three animals (9501, 9349, and 658249) died unexpectedly during the night at 9, 11, and 13 d, respectively, after having been assigned no (0) or very low (2) clinical scores. The other six animals had rapidly increased clinical scores at late checks, leading to decisions to euthanize (Fig 3F). However, post-study analysis lacked evidence clearly demonstrating infection (i.e., animals were negative for viral detection by PCR and virus titration in blood and tissues; had no evidence of infection by histopathology, Immunohistochemistry [IHC] or RNA In Situ Hybridization [ISH] in any tissues; did not seroconvert; and clinical pathology and imaging showed no signs of infection), which suggests that two of these animals (9503 and 659095) probably would have survived to the end of the study (Figs 2–8, S2, S5, S5–S22). Subsequently, the survival rate was adjusted from 25 to 41.7% (five out of 12) (AST = 11 d), and these two animals were included in the “survivor” groups for subsequent analysis.

Animals were categorized into outcome-based groups for subsequent analyses: uninfected survivors (NiV-M and NiV-B), seroconverted survivors (NiV-M), and non-survivors (NiV-M and NiV-B). Group means were compared accordingly.

Non-survivors lost a considerable amount of weight compared to survivors (Figs 3C, S5), but many animals involved in this study, including those that were uninfected, also lost weight over the course of the study. Overall, the non-survivor group had a more precipitous weight loss that correlated with disease progression, likely reflecting reduced food and water intake due to illness and increased metabolic demands from systemic infection. Non-survivors with NiV-M infection lost more weight than non-survivors with NiV-B infection likely due to a longer disease course. Weight was relatively unchanged in all survivors exposed to either NiV-M or NiV-B (Figs 3C, S5).

Animals with NiV-M infection had a transient body temperature increase to up to one degree higher than baseline at 10–14 d (Figs 3D, S6), but this decreased to sub-baseline levels at endpoint for non-survivors. Non-survivors with NiV-B infection did not develop fever over the duration of the disease, but a marked temperature drop, more than that observed in NiV-M non-survivors, was evident at the time of euthanasia. Temperature change was not remarkable in animals that survived virus exposure (Figs 3D, S6).

Respiration rates were elevated in most non-survivors with either NiV-M or NiV-B (Fig 3E). Increased respiration in NiV-M non-survivors likely reflects systemic illness and respiratory involvement during severe disease. Despite a transient fever, these animals experienced respiratory stress similar to NiV-B non-survivors, indicating that both virus strains can cause respiratory compromise.

### Clinical pathology

Complete blood counts (CBCs) were generally unremarkable in animals exposed to NiV-M, except for an elevation in white blood cells observed in non-survivors, consistent with an active viral response (Figs 4A, S7). Additionally, increased red blood cell count, hemoglobin, and hematocrit levels in these animals suggest dehydration at the terminal phase of the disease course (Figs 4A, S8–S10). As was previously identified in animals exposed to NiV-M in the context of small-particle and intermediate particle aerosols [20,21]. A precipitous decrease in platelet counts is a hallmark of lethal disease. Here, almost all non-survivors showed a rapidly developing thrombocytopenia, while those that survived did not (Figs 4A, S11). Based on serum chemistry, NiV infection induced progressive vasodilatory shock and multi-organ dysfunction in animals that succumbed. Specifically, serum chemistry analysis revealed elevated levels of glucose, blood urea nitrogen (BUN), creatinine, alkaline phosphatase, and amylase, along with decreased levels of albumin at the terminal phase of disease. These changes, observed predominantly in non-survivors, indicate systemic metabolic disturbance, multi-organ dysfunction (kidney, liver, renal), and a systemic inflammatory response. Interestingly, each of the three NiV-M non-survivors had markedly elevated amylase levels, indicating pancreatic damage (Figs 4B, S12–S17). NiV-M infection of the pancreas in AGMs has been identified previously, with evident degeneration of the islets [21]. Seroconverted survivors had serum chemistry consistent with that of uninfected animals exposed to NiV-M or NiV-B (Figs 4B, S12–S17).

Analysis of coagulation factors in NiV-B non-survivors demonstrated an increase in partial thromboplastin time (PTT) and thrombin time (TT), while fibrinogen and von Willebrand factor (VWF) antigen levels were elevated in both NiV-M and NiV-B non-survivors; these data correlated with the development of thrombocytopenia (Fig 4C). In contrast, animals with NiV infection that survived did not exhibit marked evidence of thrombocytopenia despite showing increased/decreased levels of fibrinogen and VWF antigen levels during the acute phase of disease that returned to normal during convalescence. Increased PTT and TT, mainly observed in NiV-B non-survivors, reflect disrupted coagulation pathways, suggestive of potential hemorrhagic complications, including disseminated intravascular coagulation (Fig 4C). Histopathological analysis also demonstrated clear evidence of hemorrhage in the lungs of non-survivors (Fig 8B).

### Virus dissemination

Analysis of viral RNA (vRNA) load in blood, nasal swabs, and oral swabs showed clear evidence of vRNA in non-survivors (Figs 5A–5D, S18, S19). Animals that survived and did not seroconvert had no evidence of vRNA in whole blood, serum, or swabs. Animals with NiV-M infection had low to undetectable vRNA in whole blood and serum, but many had several log_10_ gene copies of vRNA in the nasal swabs and low amounts of vRNA in oral swabs (Figs 4D, 5C, S19), supporting serological data that these animals were infected but recovered from the infection. vRNA was first detected at 3 d post-exposure in oral swabs from surviving animals, whereas in non-survivors, vRNA detection was delayed, first appearing at 6 d, and continued to replicate until study endpoint (16–20 d post-exposure). Distinct patterns of viral kinetics were observed between survivors and non-survivors: rapid viral clearance or controlled replication in survivors (by Week 2), whereas delayed and uncontrolled viral replication was associated with fatal outcome (Figs 5C, 5D, S19). These data also highlight the value of using the sampling of nasal swabs as a sensitive diagnostic tool in human cases and further demonstrate that human-to-human transmission may occur through inhalation of aerosols or droplets. vRNA was detected only in tissues of non-survivors exposed to NiV-M, with high vRNA levels present in multiple tissues, including lungs, spleens, livers, kidneys. This pattern indicates extensive viral dissemination at the time of death. In contrast, no vRNA was detected in any tissues from survivors, consistent with effective control and clearance of infection (Figs 5E, S20). Viable virus was detected by plaque assay in the lungs, kidneys, liver and spleens of non-survivors but not in the organs of any survivors (Figs 5F, S21).

In animals with NiV-B infection, RNA replication began as early as 3 d and reached peak levels by the time of death (8–14 d), as detected in whole blood, serum, and swabs. The viral load was significantly higher than that observed in animals with NiV-M infection, with no vRNA detected in survivors, indicating that high viral load, rapid replication, and failure to clear the virus contribute to the rapid progression of disease (Fig 5A–5D). vRNA was detected by qRT-PCR in all tissues tested in non-survivors, but nothing detected in survivors (Figs 5E, S20). Viable virus was detected by plaque assay only in the lungs, kidneys, liver, pancreas, or spleen of the survivors (Figs 5F, S21). These findings imply that effective viral clearance is associated with survival and uncontrolled viral dissemination contributes to disease severity and fatal outcomes.

### Host immune response to NiV-M and NiV-B

#### Serology.

Variable immune responses to NiV-M exposure were observed. Animals with NiV-M infection developed measurable immunoglobulin M (IgM), immunoglobulin G (IgG), and neutralizing antibody responses (Fig 6A). Four out of five animals, including one survivor (8671), seroconverted after exposure to NiV-M. Among the two survivors, one animal did not seroconvert, indicating that it was not infected. A lower measured dose (61.6–196.6 PFU) of virus was delivered to cohort 2 animals compared to the dose for cohort 1 (224.4–773.7 PFU), and all cohort 2 animals survived through the end of the study with no clinical signs of disease (Fig 3B, 3F). Five out of six animals in cohort 2 seroconverted (Figs 2, 6A). Among animals that succumbed, IgM responses were similar to those of survivors, and two animals developed IgG and neutralizing antibody levels comparable to survivors (Fig 6); however, IgG induction occurred several days later than in survivors (16–20 vs. 14 d), suggesting delayed or suppressed adaptive responses that provided insufficient protection against the rapidly progressing disease. In contrast, survivors maintained robust antibody levels through the end of the study. An antibody response was not detected in two surviving animals, suggesting these animals were exposed but not infected (Figs 2, 6A).

None of the animals exposed to NiV-B had evidence of serum IgM, IgG, or neutralizing antibodies, indicating that the death of three non-survivors occurred in the absence of protective antibody responses (Figs 2, 6A). The animals that succumbed did so within 14 d, so the lack of an IgG response is not surprising. However, an IgM and associated neutralizing response would have been expected in samples collected in the latter stages of the acute disease. The absence of detectable antibodies (including IgM) in animals with NiV-B infection likely reflects the combination of rapid disease progression, immune evasion by the virus, and systemic immune dysregulation, which prevented the timely development of an effective humoral response prior to death. There’s no evidence of infection in survivors because no antibody was detected, no viral replication occurred throughout the study, and no disease was identified in histopathological and molecular pathological analyses (Figs 2, 6A).

#### Immune cell dynamics following NiV-M and NiV-B exposure.

Longitudinal flow cytometry analysis of whole blood was conducted by multiparameter flow cytometry to determine if changes in immune cell populations correlated with different disease outcomes or if there was a relationship between systemic immune response and disease caused by NiV-M and NiV-B. Given the relatively small group size and variability among these outbred animals, statistical significance was not evaluated in this analysis.

Interestingly, in NiV-M-exposed animals, those that succumbed had the highest baseline of CD8 + T cell (singlets+live+CD45 + CD3 + CD8+) and B-cell (singlets+live+CD45 + CD3-CD20+) populations, while those that were infected but survived had the lowest, and uninfected animals were generally in between. In contrast, this trend reversed with the lowest baseline of CD4 + T cells (singlets+live+CD45 + CD3 + CD4+) in NiV-M-exposed animals that succumbed, and the highest baseline level in survivors seroconverted and uninfected. Decreases seen in B, T, and natural killer (NK) (singlets+live+CD45 + CD3-CD20-CD14-) cell populations in animals with acute illness that met endpoint criteria for euthanasia were followed by slight increases and a rebound at the terminal phase of disease (those with NiV-M infection); similar activity was seen survivors but not evident in animals exposed to NiV-M that remained uninfected. These data suggest that lymphocyte depletion and partial recovery may reflect strain-specific immune modulation and disease progression.

In animals exposed to NiV-B that succumbed, an elevation in baseline levels of CD4 + T cell populations was observed. These levels initially declined during the acute phase, followed by either slight increases (CD20 + B cells and NK cells) or moderate increases (CD4+ and CD8 + T cells) at the terminal phase of the disease (Fig 6B). Nevertheless, the baseline data for CD8 + T cells, CD20 + B cells, and NK cells from both animals with NiV-B infection and uninfected animals remained consistent.

#### Cytokine/chemokines/growth factors.

Plasma samples collected serially were analyzed to assess alterations in the levels of cytokines, chemokines, and growth factors throughout the progression of acute disease and the convalescence phase. Animals that met endpoint criteria and were euthanized following exposure to NiV-M had rapidly increasing levels of IL-1RA, IFN-γ, IL-15, and IL-2 (Fig 6C), similar to what was seen in animals with NiV-M infection following intermediate particle exposure [23]. Animals that survived infection also had transiently increased levels of IL-1β, IFN-γ, IL-15, G-CSF, and IL-2, each of which returned to baseline at ≈10–14 d post-exposure, suggesting regulation of a systemic inflammatory response (Fig 6C).

NiV-M survivors exhibited a sustained elevation in G-CSF and IL-2 levels, indicating an activation or expansion of granulocyte and T-cell populations, respectively, throughout the convalescence period. In animals exposed to NiV-M but not infected, there were no alterations in cytokine or chemokine levels, except for G-CSF. The growth factor exhibited an increase 10–14 d post-exposure, subsequently returning to baseline levels, and then increased again at the conclusion of the study.

Analogous to animals with NiV-M infection, those that succumbed to NiV-B infection exhibited a rapid elevation in IL-1RA, IL-6, IFN-γ, GM-CSF, IL-10, IL-15, MCP-1, and MIP-1β during the terminal phase of the disease (Fig 6C), in contrast to the uninfected animals, for which no notable changes were observed over time in most cytokines (except IL-1β and IL-2).

### Imaging

The use of imaging has been crucial for demonstrating disease progression in this model and has been valuable for understanding the disease process in the absence of clear clinical signs, particularly in animals that developed neurological disease.

Computed tomography (CT) imaging was primarily used to evaluate lung disease, while brain MRI was used to assess the neurological consequences of virus exposure. Imaging data from one of the initial NiV-M cohorts has been previously published [21,22]. Representative serial longitudinal imaging scans (chest CT/brain MRI) illustrating these findings are presented, with corresponding interpretations provided thereafter (S1–S4 Figs).

#### Brain MRI analysis.

Brain MRI was reported previously in NiV-M-exposed cohort 1 animals [22]. In brief, brain lesions were identified in four out of five animals in NiV-M-exposed animals using both histopathology and MRI. One animal in this cohort (8212) did not develop clinical signs of disease, showed no brain lesions on MRI, and tested negative for NiV by PCR, IHC, and all other viral detection assays (Figs 2, 5, S1A, S18–S20, S22A). The data presented here demonstrate that this animal was uninfected. In cohort 2, one out of six animals exposed to NiV-M showed very subtle fluid-attenuated inversion recovery (FLAIR) hyperintense brain lesions by MRI, which resolved over time, and the animal was lesion-free at the end of the study (S1B Fig).

Due to rapid disease progression, previous data, and study design, images were attained after infection presentation in four NiV-B-exposed cohort 1 animals and one out of six in cohort 2. In cohort 1, only one animal that developed clinical infection was imaged at 10 d; no abnormal findings were present in the brain MRI. Three other animals from cohort 1 were imaged; however, they had not developed clinical infection and had normal MRI scans. In cohort 2, the one animal that was imaged at 14 d had not developed clinical disease. None of the other five animals, including two found to have had non-acute brain histological abnormalities, was imaged after infection (Figs 2, 3, S2).

#### Lung CT analysis.

In animals exposed to NiV-M, either prominent, diffuse opacities or small focal opacities were observed in four out of five animals in cohort 1, with two animals showing pleural effusions. In cohort 2, three out of six animals exposed to NiV-M exhibited opacities, but none showed pleural effusions. Opacities resolved over several days in one out of five animals in cohort 1 and in all three animals in cohort 2, with these animals surviving until the end of the study. One surviving animal in cohort 1 showed mild opacities intermittently throughout the study that resolved by 22 d (S3 Fig).

CT scans showed patterns of alveolar consolidation, interstitial infiltrates, and evidence of edema in NiV-M-exposed animals. In animals exposed to NiV-B, three out of six animals in cohort 1 showed mild to diffuse opacities in the lungs associated with NiV-B exposure, while one animal (09357) exhibited mild opacities likely unrelated to NiV-B exposure. Opacities resolved over time in one out of three animals. In cohort 2, four out of six NiV-B-exposed animals showed mild to moderate diffuse opacities and pleural effusions, most of which remained unresolved until euthanasia criteria were met (S4 Fig).

To evaluate pulmonary disease progression, quantitative group analysis of lung CT data showing dynamic percent change in lung hyperdensity (PCLH) was conducted longitudinally by serial chest CT imaging in animals exposed to either NiV-M or NiV-B (Fig 7). PCLH quantifies parenchymal changes consistent with inflammatory/infectious process of the lung over the course of disease. Animals with NiV-B infection that met endpoint criteria and were euthanized (*n* = 7) exhibited marked increases in PCLH, with peak values exceeding 350%. In contrast, animals with NiV-M infection that met endpoint criteria and were euthanized (*n* = 3) demonstrated peak PCLH values of ≈200%. Minimal changes in PCLH were observed among uninfected animals (NiV-M: *n* = 2; NiV-B: *n* = 5) and seroconverted survivors (NiV-M: *n* = 6) over the study period. Statistical analysis revealed a significant difference in peak PCLH between NiV-B-exposed animals that were euthanized after meeting endpoint criteria and all other groups, indicating that more severe pulmonary disease occurred in NiV-B-exposed animals that succumbed to the infection.

### Pathology

Among animals exposed to NiV-M or NiV-B, the constellation of gross and histologic lesions was consistent with previously reported NiV infection in the AGM model [21,24]. However, in the present study, there were several notable differences between lesions seen with NiV-M and NiV-B, as outlined below (Figs 8, S22).

For both virus strains, lymphoid tissues were most consistently affected, particularly the splenic white pulp and peripheral lymph nodes. Within these tissues, lymphocyte necrosis and depletion of follicular germinal centers were seen in seven out of 12 animals exposed to NiV-B and five out of 11 animals exposed to NiV-M. This lymphoid depletion was frequently accompanied by syncytial cell formation in the marginal zone adjacent to the lymphoid follicles, with occasional hemorrhage of the lymphoid follicles, with greater incidence and severity in animals exposed to NiV-B (Fig 8A: a–d, 8E).

The lungs were also consistently affected in both NiV-M and NiV-B exposure groups. Interstitial pneumonia, predominantly composed of non-suppurative inflammation in the interstitium, was seen in four out of 12 animals exposed to NiV-B and in four out of 11 animals exposed to NiV-M. This inflammation was variably accompanied by alveolar hemorrhage, alveolar necrosis, fibrin, hyaline membrane deposition, and syncytial cell formation in pneumocytes and endothelial cells. Notably, the presence of significant amounts of fibrin and hyaline membrane formation was limited to animals exposed to NiV-B (5/12) and was not appreciated in any of the 11 animals exposed to NiV-M. Similarly, alveolar hemorrhage was more commonly seen in animals exposed to NiV-B (5/12) than those exposed to NiV-M (2/12) (Fig 8B: e–h, 8E).

Within the central nervous system, appreciable histopathologic brain lesions were only noted in two animals exposed to NiV-M. Five out of 11 had encephalitis, characterized by random foci of lymphocytes, plasma cells, and microglia surrounding areas of neuronal necrosis, edema, and myelin degradation in the white and gray matter, most commonly affecting the ventral midbrain around the olfactory tubercle, nucleus accumbens, and ventral pallidum. Two out of 12 animals exposed to NiV-B had rare individual neuron necrosis randomly within the cerebrum, but the lack of surrounding inflammation or viral protein/genomic material via immunohistochemistry makes it difficult to attribute these lesions to NiV-B exposure and suggest pre-existing damage (Figs 8C: i–m, 8E, S22).

Histologic lesions attributable to NiV exposure were also encountered in the urinary bladder, which also showed mucosal inflammation, hemorrhage, and thrombosis within the submucosa, and ulceration of the urothelium with occasional urothelial syncytial cell formation. These lesions were seen in two out of 12 of the animals exposed to NiV-B and one out of 11 in animals exposed to NiV-M. The remainder of lesions in animals exposed to either virus strain was fairly non-specific and are likely only secondarily associated with protracted NiV infection (such as vacuolar hepatocellular degeneration in the liver secondary to decreased dietary intake) or represent common background findings in mature AGMs (Fig 8D: n and o, 8E).

Viral detection across all tissues using immunohistochemistry [IHC] (NiV-M, NiV-B, cohort 1 animals) and RNA In Situ Hybridization [ISH] (NiV-M, NiV-B, cohort 2 animals), respectively (S22 Fig). NiV antigen (S22A Fig) was detected only in almost all non-survivors of both NiV isolates and was widespread across respiratory, lymphoid, visceral, and central nervous system tissues. In Cohort 2, viral RNA (S22B Fig) was also detected exclusively in non-survivors, with broad distribution across the same organ systems. All survivors were negative by both methods, consistent with effective viral clearance and the absence of systemic infection at necropsy. These findings support the conclusion that uncontrolled viral dissemination is a key feature of fatal disease.

## Discussion

The objective of the study described here was to identify a NiV infection model that recapitulated the disease seen in humans. A series of experiments was performed using increasingly larger aerosol particle sizes to mimic inhalational exposure, leading to a range of disease phenotypes [20–23]. In the work presented here, to better replicate human exposure via inhalation of droplets, a relatively low target dose (≈500 PFU) with a larger particle size (≈12 μm) was used to expose AGMs to either NiV-M or NiV-B. As published previously [22], the large-particle aerosol NiV-M exposure model successfully replicates natural respiratory exposure in humans, especially in regard to the development of neurological disease features, which recapitulate human central nervous system (CNS) disease. The work described here expands upon initial studies, providing a more comprehensive analysis of the overall host response to NiV-M infection, and compares animals similarly exposed to NiV-B. The findings from this work demonstrate clear differences in disease dynamics between the two NiV strains. NiV-M infection can lead to a heterogeneous disease spectrum: 27% (3/11) of animals developed progressive neurologic disease, met endpoint criteria, and were euthanized at 16–20 d; six animals were asymptomatic survivors, and two remained uninfected despite confirmed exposure. Neurologic involvement, including encephalitis and vasculitis, was evident on MRI/histopathology in infected animals (non-survivors and survivors) but not necessarily by cage-side observation. In contrast, NiV-B infection led to a more severe disease phenotype, with 58.3% (7/12) of animals meeting endpoint criteria and being euthanized due to a rapidly progressing respiratory disease at 8–14 d. The remaining five animals were uninfected despite having been exposed, exhibiting minimal clinical signs, no serologic responses, and no virologic evidence of infection. Pulmonary pathology was more extensive in animals with NiV-B infection, while neurologic manifestations were less frequent or absent.

In this study, the fatality rate following NiV-M and NiV-B exposure is relatively lower than previously reported in AGMs exposed via concurrent IT/IN or small-particle aerosol routes [19,20,25–27]. This may more accurately reflect the heterogeneity observed in human disease, particularly with NiV-B, which did not result in uniformly fatal outcomes in our study. Mire et al. previously compared NiV-M and NiV-B infection in AGMs using a concurrent IN/IT exposure that resulted in a rapidly progressing disease in animals with NiV-B infection and a longer disease course in those with NiV-M infection [19]. The overall survival and disease course results reported here are similar to those from Mire et al., who reported a more aggressive disease course and uniform lethality for NiV-B compared to NiV-M; however, that study lasted only 15 d, which means that, based on our experience, the experiment was terminated before evidence of neurologic disease would become apparent. Additionally, Mire et al. used concurrent IN/IT routes at higher doses (≈10^5–10^6 PFU), which likely promoted systemic dissemination and resulted in a more uniform onset and progression of disease before the development of a protective host response.

Importantly, our findings are consistent with recent data from the U.S. Centers for Disease Control and Prevention (CDC), which independently demonstrated that NiV-M and NiV-B differ in virulence and disease presentation. A large-scale comparative analysis in hamsters [28] showed that NiV-B produced higher lethality and more severe respiratory disease following intranasal exposure, whereas NiV-M was more lethal via intraperitoneal infection, indicating strain- and route-dependent differences in pathogenesis. These CDC findings support that the two major NiV lineages possess distinct biological properties that shape disease outcome beyond the influence of experimental dose or route and is further supported by previous work using a combined intranasal/intratracheal exposure in AGMs that showed clear differences is disease caused by the two NiV isolates [19]. Our results in AGMs exposed under standardized aerosol conditions reinforce this conclusion, demonstrating that NiV-B drives a more acute respiratory pathology, whereas NiV-M more often progresses toward neurologic involvement and vasculitis. Recognizing these intrinsic differences is critical when interpreting animal data and selecting challenge strains for vaccine or therapeutic testing.

By contrast, our large-particle aerosol model introduced more variability in virus deposition and host response, enabling the observation of a broader range of disease outcomes, including survival and the absence of infection, which better represents natural exposure scenarios. These data also suggest that large particles, or droplet exposure in the case of human-to-human transmission, are less efficient and may explain why human-to-human transmission is not typical.

The differences in disease outcome observed in our study may also reflect variations in aerosol particle size, dose, and deposition efficiency. The use of a relatively low target dose (≈500 PFU) was intended to better replicate the likely exposure levels experienced during natural human infection. Work by Clayton et al. [29] described transmission studies in which direct interaction between exposed and naïve ferrets did not result in transmission to the naïve ferrets. However, IN exposure to secreted oronasal fluid led to infection. The Clayton study and the work described here suggest that human-to-human transmission may occur through inhalation of droplets rather than direct contact with fomites. In real-world settings, NiV transmission typically occurs through inhalation of droplets, contact with contaminated surfaces, or zoonotic spillover, all of which are expected to involve relatively low doses of virus compared to high-dose laboratory inoculations. Nikolay et al., found that increased transmission correlated with infected patients who had trouble breathing and duration of close contact, which would necessarily increase the risk of infection [30]. These data suggest an increased likelihood of aerosol, or droplet, creation through forceful breathing or coughing, and continued low-dose exposure led to virus transmission to close contacts. Therefore, mimicking these lower-dose exposures is essential for capturing the full spectrum of disease outcomes, including subclinical infection, and survival with or without seroconversion. Previous high-dose studies [19,29] showed consistent fatality and could not reflect the variability observed in natural infections. By using a lower dose with large-particle aerosol exposure, our approach achieved a wider spectrum of disease manifestations: fatal, non-fatal, and abortive infections that are representative of the heterogeneity seen in human NiV outbreaks.

Although all animals received a standardized dose, the effective dose at the cellular level likely varied due to deposition efficiency and individual host factors. In this study, we did not find a correlation between particle size (range 11–14.4 µm) and fatality across the strains, indicating that within this size range, particle size alone may not be a determining factor for disease outcome. However, we observed that the relatively lower dose used in the second cohort (<200 PFU) somehow contributed to survival in NiV-M-exposed animals. However, no apparent effect on outcomes in NiV-B infection was found, which was consistent with previous reports [19,25] that severity and duration of the disease course for NiV-B-exposed animals were not associated with the challenge dose. Additionally, neither dose nor particle size appeared to influence the outcome in uninfected survivors. These findings differ from previous high-dose studies (e.g., Mire et al. and Clayton et al. [19,29]), which reported more uniform fatality and did not account for the variable host responses seen at lower, more relevant exposure levels. Nonetheless, our results are consistent with prior reports, indicating that NiV-B causes a more rapidly progressing and severe respiratory disease compared to NiV-M.

The large-particle aerosol exposure (≈12 μm) used here likely favored deposition in the upper respiratory tract, where infection control is more dependent on local mucosal immunity, including innate antiviral responses, such as type I/III interferons and resident immune cells. This may partially explain why some animals were able to clear the virus early or resist infection altogether despite confirmed exposure and suggests that respiratory protection is critical for preventing transmission. In contrast, previous studies using smaller aerosol particles (<5 μm) or direct IT/IN delivery likely promoted deeper pulmonary deposition, bypassing mucosal defenses and facilitating more uniform and severe disease outcomes [18–20,31].

Weight loss closely correlated with disease severity, with greater and more sustained loss observed in NiV-M non-survivors, likely reflecting the longer duration of disease. This pattern matches earlier studies showing that steady weight loss is a sign of worsening infection [19,32]. Body temperature increased briefly before dropping at the point animals met endpoint criteria for euthanasia, something also seen in other models. In contrast, animals with NiV-B infection failed to develop measurable fever but exhibited an abrupt temperature drop at the point of meeting endpoint criteria for euthanasia, suggestive of rapid systemic failure, perhaps driven by overwhelming inflammatory responses and vascular leakage. Elevated respiratory rates in terminal animals from both groups reinforce the presence of pulmonary pathology, as previously described in AGM and ferret models [19,29] and further support the role of respiratory compromise in NiV pathogenesis. One animal (08168) developed an acute respiratory syndrome necessitating euthanasia on 5 d. The sudden onset of symptoms may reflect rapid pulmonary involvement, but secondary factors such as gastric distension potentially impairing ventilation cannot be excluded. Additional physiological monitoring in future studies may help clarify the underlying cause of such abrupt clinical deterioration. Two NiV-B-exposed animals were euthanized based on clinical signs (low clinical scores [3 and 5]) suggestive of infection, due to concerns about rapid disease progression and the risk of losing animals unexpectedly. However, subsequent virological, serological, imaging, and pathological analyses confirmed that there was no evidence that these animals were infected. Indeed, AGMs do not show behavioral responses to experimental treatment, making interpretations during cage-side observations more difficult. These challenges underscore the importance of adopting integrated, multimodal decision-making, such as combining clinical observations with real-time viral load, blood testing, or imaging data to determine humane endpoints.

CBCs were largely unremarkable in uninfected animals but showed patterns consistent with acute viral infection in animals that succumbed. Specifically, leukocytosis, and some lymphopenia. These findings align with previous reports of systemic inflammation during NiV infection [19,20,33]. Rapid-onset thrombocytopenia was a consistent and notable feature in nearly all non-survivors (90%; Fig 3), demonstrating it as a hallmark of fatal disease, as also observed in ferret and hamster models [34,35]. Although D-dimer is a useful marker for confirming DIC, we did not have sufficient stored plasma of adequate quality from all animals to perform this assay retrospectively. Its quantification was therefore not conducted in this study and represents a limitation in confirming the extent of coagulation dysfunction, which we plan to address in future studies.

In this study, euthanasia decisions were, in some cases, influenced by the observation of a rapid drop in platelet concentration, particularly in the NiV-B cohorts, in which three animals succumbed to disease with no warning, and others presented with severe disease signs after having no clinical score in the previous cage-side check. While thrombocytopenia was considered a potential indicator of severe disease progression, not all non-survivors exhibited a platelet drop, and not all animals with platelet declines progressed to terminal disease (Fig 3). This introduces a potential bias and concern that animals showing early signs of thrombocytopenia may have been euthanized preemptively to prevent unscheduled deaths and ensure timely sample collection. As a result, some animals that might have otherwise survived or experienced a slower disease course may have been classified as having severe or fatal disease. This bias should be taken into consideration when interpreting disease outcomes and comparing pathogenesis across groups, particularly when evaluating early biomarkers or correlates of disease severity.

Serum chemistry analyses revealed elevated glucose, BUN, creatinine, and amylase, along with decreased albumin, indicating multi-organ dysfunction. Animals with NiV-M infection showed evidence of pancreatic injury, consistent with prior findings in both human and animal studies [21,36]. In contrast, animals with NiV-B infection demonstrated more severe systemic effects, including signs of shock, renal injury, and hepatic compromise, which is similar to what’s been reported in human NiV-B outbreaks [19]. Coagulation studies showed elevated PTT, TT, fibrinogen, and VWF antigen, suggesting significant disruption in hemostasis and potential hemorrhagic complications, consistent with vascular damage previously reported in both NiV-M and NiV-B infections [19,37].

Analysis of vRNA load in whole blood, nasal swabs, and oral swabs revealed clear distinctions between survivors and non-survivors. In NiV-B-exposed non-survivors, high levels of vRNA were detected in all sample types, while survivors and seronegative animals had no detectable vRNA. Among animals with NiV-M infection, survivors typically exhibited transient vRNA in nasal swabs with low to undetectable levels in blood and oral swabs, consistent with localized upper respiratory tract infection and effective immune clearance. This pattern aligns with previous findings in AGMs exposed to NiV‑M via IT, oral, or other routes (Fig 4 and [25,32]). In contrast, non-survivors exposed to NiV-M showed delayed vRNA emergence (at 9–10 d), with increasing levels until study endpoint, suggesting progressive systemic spread. In animals with NiV-B infection, vRNA was detected as early as 3 d and peaked rapidly. High viral loads were found in blood, swabs, and most visceral tissues, with viable virus confirmed via plaque assay in the lungs, liver, kidney, pancreas, and spleen, consistent with severe systemic infection. The rapid and widespread replication of NiV-B corroborates its higher virulence and aligns with previous findings of aggressive replication kinetics and host immune evasion [18,19].

Serological responses also differed significantly between the two strains. Animals with NiV-M infection that survived developed strong IgM, IgG, and neutralizing antibody responses (Fig 5A), whereas non-survivors had delayed or insufficient IgG responses despite detectable IgM and neutralization activity, suggesting that early antibody production may contribute to protection but is not always sufficient to prevent fatal outcomes. Serum from the NiV-M survivors was found to contain measurable NiV-specific IgG and IgM antibodies within 2 weeks after exposure, and the clearance of NiV from blood and swabs at 2 weeks indicated the elicitation of virus-specific antibody in response to the infection. NiV-M survivors had elevated counts in B lymphocytes in ≈2 weeks, correlating with the generation of NiV-specific IgM and IgG antibodies (Fig 5B). Altogether, these data demonstrate that an adaptive humoral immune response afforded protection against NiV-M infection in both acute and convalescent phases. In contrast, NiV-B non-survivors and survivors exhibited no measurable IgM, IgG, or neutralizing antibodies before the endpoint. Given the disease course (time to death 8–14 d), the absence of humoral responses likely reflects rapid progression and potential immune suppression, possibly mediated by viral interferon antagonists such as the W and V proteins [38]. This supports prior evidence that NiV-B is more adept at evading or dampening host immunity, impairing the development of a protective response in time to prevent death. Notably, NiV-B survivors showed no seroconversion, no detectable vRNA, and no histopathologic evidence of infection, further suggesting that these animals were not productively infected despite exposure.

Flow cytometry revealed distinct immune cell profiles associated with different disease outcomes and viral strains. In animals with NiV-M infection, non-survivors had higher baseline levels of CD8 + T cells and B cells, while survivors showed the lowest levels of these populations. Interestingly, CD4 + T cells followed an opposite trend, being lowest in non-survivors and highest in survivors. These baseline differences may reflect pre-existing immune readiness or differential immune regulation, as previously observed in viral infection models in which heightened baseline CD8 + T cell responses correlated with immunopathology rather than protection [39]. During acute illness, all infected animals, regardless of outcome, showed the trend of declines in circulating B cells, T cells, and NK cells, consistent with lymphoid depletion and systemic inflammation, similar to findings in our previous study [23]. In one survivor, these cell populations partially rebounded during convalescence, indicating a restoration of immune homeostasis. In animals with NiV-B infection, fatal cases had higher baseline CD4 + T cells, which sharply declined during peak illness and rebounded, particularly CD4 + T and NK cells, toward the terminal phase. These data may indicate a delayed but overwhelming immune response, characteristic of cytokine-driven immunopathology. Uninfected animals maintained stable immune cell profiles throughout the study, supporting the hypothesis that mucosal or innate immune barriers may have prevented systemic spread and immune perturbation. These findings collectively suggest that immune cell dynamics are influenced not only by the virulence of the infecting strain but also by host baseline immune states, and they may be predictive of disease outcome. Additional studies could confirm these patterns and determine if they are statistically significant or directly related to disease progression.

Cytokine profiling highlighted notable differences in host immune activation between NiV-M and NiV-B infections. In animals with NiV-B infection, we observed an early and robust pro-inflammatory cytokine surge characterized by elevated IL-6, TNF-α, IFN-γ, IL-1RA, and MIP-1β levels, markers commonly associated with cytokine storm syndromes and systemic immune dysregulation. This acute cytokine elevation likely contributed to the rapid disease progression and extensive tissue damage observed in NiV-B fatalities. In contrast, animals with NiV-M infection exhibited a more delayed and moderate cytokine response. While increases in IFN-γ, IL-15, and IL-1RA were still noted [40], especially in non-survivors, the overall inflammatory profile was less intense than that seen with NiV-B. This attenuated response may reflect the activity of NiV-M’s accessory proteins, particularly the W and V proteins, which are known to antagonize interferon signaling by targeting STAT1 and STAT2 pathways [38]. These differences may allow NiV-M to evade early immune detection more effectively, resulting in a more protracted disease course with delayed immune activation. Collectively, these findings support observations that NiV-B induces a more aggressive systemic inflammatory response, whereas NiV-M may subvert host defenses to establish a slower, yet still potentially fatal, infection. The divergent cytokine profiles likely play a key role in the distinct clinical and pathological trajectories observed between these two strains.

MRI and CT imaging revealed distinct patterns of brain or lung involvement between NiV-M and NiV-B infections. Neurological pathology was primarily observed in animals with NiV-M infection, consistent with its reported neurotropism. MRI identified multifocal brain lesions in several animals, correlating with histopathological findings, such as non-suppurative encephalitis, gliosis, and perivascular cuffing. Some lesions were detected even in animals that survived infection, suggesting subclinical CNS involvement and highlighting the sensitivity of MRI in identifying early or mild neurologic changes. These findings align with those of prior studies, including cohort 1 of the current study, published by Lee et al. [22], which demonstrated MRI-detectable CNS abnormalities in AGMs with NiV-M infection. Histopathological studies further supported these observations, with encephalitis and ischemic changes found in brain tissue from both fatal and surviving cases [41]. In contrast, animals with NiV-B infection exhibited minimal neurologic involvement by histopathology. Only isolated acute microinfarctions were observed in non-survivors that met endpoint criteria and were euthanized early, with no MRI data available due to the rapid disease course. The apparent absence of CNS pathology is likely due to the more aggressive and systemic nature of NiV-B, which results in earlier fatality and may not allow time for viral neuroinvasion.

Lung CT imaging was especially informative in animals with NiV-B infection, for which it revealed extensive pulmonary involvement consistent with severe respiratory disease. CT scans showed patterns of alveolar consolidation, interstitial infiltrates, and evidence of edema, supporting histological findings of pneumocyte necrosis, hemorrhage, and fibrin deposition. These imaging features correlate with human CT presentations during severe NiV infections and affirm the utility of CT as a non-invasive tool for monitoring disease progression without serial euthanasia of animals. In animals exposed to NiV-M, lung CT abnormalities were more variable and often milder, reflecting the more heterogeneous respiratory involvement compared to NiV-B. Together, these imaging findings underscore the differing pathogenesis of NiV-M and NiV-B: NiV-M is more prone to CNS dissemination with slower progression, while NiV-B produces rapid, fatal respiratory disease with widespread pulmonary pathology and minimal neurologic impact.

As with previous studies, there is evidence of vascular breakdown and hemorrhage in non-survivors exposed to either NiV-M or NiV-B. Hemorrhage and vasculitis were major pathologic features in both NiV-M and NiV-B infections, but they manifested differently. In animals with NiV-M infection, vasculitis was widespread, involving small to medium-sized vessels across multiple organs, including the brain, lungs, and liver. This is consistent with previous studies showing that NiV-M induces endothelial damage and immune-mediated vascular inflammation, a key hallmark of NiV pathogenesis [25,41]. Vascular lesions in NiV-M cases often included perivascular cuffing, endothelial syncytia, and inflammatory infiltrates, which likely contributed to the development of encephalitis and other organ damage seen in these animals. In animals with NiV-B infection, hemorrhagic lesions were more prominent and diffuse, especially in the lungs. Histopathology revealed alveolar hemorrhage, vascular congestion, and fibrin thrombi, indicating severe pulmonary vasculopathy and coagulation disturbances, which correlate with the elevated PTT, and TT values found via clinical pathological analysis. These features suggest acute endothelial dysfunction and a possible disseminated intravascular coagulation-like phenomenon driving rapid respiratory failure and systemic collapse. Although vasculitis was present in NiV-B-exposed animals, it was less organized and more secondary to the intense vascular injury and tissue necrosis caused by the infection’s aggressive course. Altogether, while vasculitis is a defining feature of NiV-M pathogenesis, hemorrhagic lung disease dominates in NiV-B, reinforcing their distinct pathogenic profiles. These differences are crucial when modeling human disease, because neurological complications and systemic vasculitis are common in NiV-M human cases, whereas severe respiratory symptoms and shock-like states are often reported in NiV-B outbreaks.

Overall, this study underscores the higher virulence of NiV-B compared to NiV-M, while highlighting the critical influence of exposure route and dose on disease trajectory. The marked heterogeneity observed, particularly among animals with NiV-M infection, demonstrates the utility of large-particle aerosol models in capturing a broad spectrum of clinical outcomes, including both survivable and lethal phenotypes. This model closely mimics natural human transmission via inhalation of droplets and thus is well-suited for evaluating host susceptibility, immune correlates of protection, and the efficacy of medical countermeasures under physiologically relevant conditions.

Importantly, our use of 11–12 AGMs per group provides one of the most comprehensive comparative analyses to date, enhancing the statistical power and translational relevance of our findings. This study contributes to the field by: (1) establishing a robust and representative animal model of NiV-M and NiV-B infection; (2) identifying potential immune correlates of protection, such as early viral clearance and humoral responses; and (3) revealing strain-specific differences in pathogenesis, immune evasion, and tissue tropism that are essential for guiding future vaccine and therapeutic development. Together, the insight provided by these interpretations fills a key translational gap and provides a strong foundation for advancing NiV preparedness.

## Materials and methods

### Ethics statement

Work with nonhuman primates was conducted at the Integrated Research Facility at Fort Detrick (IRF-Frederick), which is accredited by the Association for the Assessment and Accreditation of Laboratory Animal Care. The IRF-Frederick is part of the National Institutes of Health (NIH) National Institute of Allergy and Infectious Diseases (NIAID). All animal procedures were approved by the NIAID Animal Care and Use Committee and conducted in compliance with the Animal Welfare Act regulations, Public Health Service policy, and the Guide for the Care and Use of Laboratory Animals (Eighth Edition). All work with nonhuman primates was done following the recommendations of the Weatherall report.

This work was approved by the National Institutes of Health Institutional Biosafety Committee and the Dual Use Research of Concern (DURC) Institutional Review Entity (IRE).

### Animals

Wild-caught AGMs (*Chlorocebus sabaeus*) of Caribbean origin were purchased from PrimGen (Hines, IL, USA). Animals were identified for inclusion based on similarity of size with equal numbers of males and females in each cohort (*n* = 12 per NiV isolate). Work was divided into four separate experiments in which group sizes (*n* = 6 per experiment) were limited (up to *n* = 3 per d) due to clinical imaging requirements associated with this study. AGMs (3.5–8 kg) were pre-screened for NiV antibodies (using enzyme-linked immunosorbent assay [ELISA] and/or neutralization assay) upon arrival to ensure suitability for the study. Animals were group-housed before being assigned to the study and were singly housed during the conduct of the study. At all times, animals were provided with appropriate enrichment, including but not limited to polished steel mirrors and durable toys. Animals were anesthetized under biosafety level 4 (BSL-4) standard protocols before all procedures, including virus exposure, imaging, and collection of blood to minimize stress to the animals. Animals were observed after anesthesia to ensure complete recovery.

### Viruses and cell culture

The NiV-M was isolated from a fatal human case in 1998 [42]. Virus stock was obtained from a collection housed at the U.S. Army Medical Research Institute for Infectious Diseases and has a documented history that includes three passages in Vero E6 cells, one passage in Vero cells, and two additional passages in Vero E6 cells after receipt in our facility. NiV-B (prototype 200401066) was provided by the U.S. Centers for Disease Control and Prevention. This isolate has one documented passage in Vero E6 cells prior to arrival in our facility, with two additional passages in Vero E6 cells at the IRF-Frederick.

The stock virus sequence information for NiV-M (IRF0160) is available through GenBank (Accession #KY425646.1) and is consistent with the previously published sequence for this virus (Accession #AF212302). The sequence for NiV-B (IRF0284) has been submitted to GenBank (pending acceptance) and is consistent with the previously reported sequence for this isolate (#AY988601).

Vero E6 cells (BEI Resources, Manassas, VA, USA) were maintained at 37°C in 5% carbon dioxide in α-MEM w/GlutaMAX (Thermo Fisher, Waltham, MA, USA) and containing 10% fetal bovine serum (FBS) (Sigma-Aldrich, St. Louis, MO, USA). All work with viable NiV was performed in BSL-4 containment at the IRF-Frederick.

### Animal study design

A total of 24 adult AGMs (assigned to two groups of 12, each of which contained two cohorts of six) were exposed to either NiV-M or NiV-B at a target dose of ≈500 PFU (confirmed by back-titration). Animals were monitored at least twice daily after virus exposure for clinical signs of disease. Physical exams, blood collection, and swab (nasopharyngeal and oropharyngeal) sampling were performed at two pre-exposure (baseline) time points; at 3, 6, 10, 14, 18, 24, 31, 38, and 45 d post-exposure; and euthanasia (56–63 d). Whole-body CT and brain MRI (with/without contrast) were acquired; in cohorts 2, 3, and 4, early-phase MRI started at 10 d (when brain abnormalities were expected) to mitigate anesthesia-related complications (gastrointestinal ileus and distension) noted in cohort 1. Due to minor discrepancies in imaging/sampling timelines across cohorts, group analyses were aligned by study week after the acute phase. This adjustment facilitated data interpretation and comparison of NiV-M and NiV-B pathogenesis under consistent exposure and monitoring conditions (Fig 1).

### Aerosol exposure

Animals in each cohort (*n* = 3 per exposure per day) were exposed to a target dose of 500 PFU by large-particle (≈10–12 µm) aerosol containing NiV-M or NiV-B as described previously [22]). Large-particle aerosols deposit primarily within the nasopharyngeal region of the respiratory tract, avoiding deep lung penetration and alveolar deposition [43]. On the days of exposure, the stock virus was thawed, pooled, and diluted to create an aerosol starting concentration, which remained on wet ice for the duration of the experiment. Animals were given glycopyrrolate (0.006 mg/kg via the intramuscular [IM] route) to reduce nausea and anesthetized with ketamine (15.0 mg/kg IM) and Telazol (2.0 mg/kg IM) to ensure a steady-state plane of anesthesia for the duration of the aerosol exposure and plethysmography acquisition. After anesthesia administration and before exposure, plethysmography acquisition was performed to obtain breaths per minute, inhaled volume (mL), and inhaled minute volume (L/min). Values were gathered using an Accutach pneumotach flow sensor (BB Medical Technologies, CA, USA), attached to a small canine anesthesia mask (Patterson Veterinary, CO, USA), via the Hans Rudolph system (Hans Rudolph Inc., KS, USA) using the SmartLab with Insight software. Large-particle aerosols were generated using a 60.0-mL syringe with a liquid feed rate of 1.0 mL/min of diluted virus onto a ≈ 40,000.0-RPM polyetheretherketone (PEEK) spinning cup centered inside a stainless-steel outer plenum. An aerodynamic particle sizer (TSI Incorporated, Shoreview, MN, USA) verified the particle size and distribution, displaying a real-time measurement of mass median aerodynamic diameter, total particle count, total concentration, and geometric standard deviation for each aerosol administration session. Gelatin filters (Sartorius Stedim Biotech, Goettingen, Germany), placed on a mesh screen and contained within a stainless-steel biosampler (In-Tox Products, Clinton, MS, USA), were attached to the exposure chamber, operating at a flow rate of 6.0 L/min and collecting viral material from each aerosol session for back-titration and virus quantification via plaque assays. A presented or estimated inhaled dose was calculated using a simplified calculation: *D* = *R* × *C*exp × *T*exp, where *D* is the presented or estimated inhaled dose (PFU), *R* is the respiratory minute volume (L/min), *C*exp is the aerosol concentration (PFU/L) from the chamber, and *T*exp is the duration of the exposure (min). This formula has been outlined previously [44].

### Clinical observations

Physical exams included general visual inspection of each animal, determination of body weight, temperature, blood pressure, respiration rate, heart rate, and oxygen saturation (pulse oximeter). During clinical observations, animals were assessed and assigned clinical scores based on a 10-point scale outlined in the approved animal study protocol; parameters included temperature, responsiveness, recumbency, and respiration. Animals meeting endpoint criteria were euthanized per the approved protocol; survivors were euthanized at ≈56–63 d.

### Clinical pathology

#### Blood analyses.

Whole blood samples were collected into ethylenediamine tetraacetic acid (EDTA) tubes and analyzed using a Sysmex XT-2000iV automated hematology analyzer (Sysmex, Kobe, Japan). Parameters measured included white blood cell count, red blood cell count, hemoglobin, hematocrit, platelet count, and differential leukocyte counts.

#### Serum chemistry.

Serum was isolated by centrifugation of whole blood collected in serum separator tubes. Biochemical analyses were performed using a Piccolo Xpress Chemistry Analyzer (Abaxis/Zoetis, NJ, USA) with the Piccolo General Chemistry 13 panel. The following analytes were measured: alanine aminotransferase, aspartate aminotransferase, alkaline phosphatase, gamma-glutamyltransferase, albumin, total protein, globulin, amylase, BUN, creatinine, glucose, total bilirubin, and electrolytes.

#### Coagulation assays.

Plasma was collected into 3.2% sodium citrate tubes, centrifuged, and stored at appropriate temperatures until analysis. Activated PTT, TT, fibrinogen, and VWF antigen were measured using an automated coagulation analyzer (Siemens Sysmex CA-660) following the manufacturer’s protocols.

### Viral replication

PCR samples were inactivated in TRIzol LS (Thermo Fisher) per in-house BSL-4 safety protocols. Total RNA was isolated using the QIAamp Viral RNA Mini Kit (Qiagen, Germantown, MD, USA). Briefly, 70 µL of TRIzol LS inactivated sample was added to 280 µL of QIAGEN Buffer AVL containing carrier RNA. The sample was eluted in 70 µL of Buffer AVE, aliquoted, and frozen until assayed. Sample viral load was then measured using an in-house quantitative reverse-transcription PCR assay [45] for NiV and reported as viral RNA copies (log_10_) per mL of sample.

Viral titer was determined through plaque assay following an in-house standard operating procedure. Briefly, samples were serially diluted in Dulbecco’s Modified Eagle’s Medium (Thermo Fisher) with 5% heat-inactivated FBS (Sigma-Aldrich) and antimycotic and analyzed in triplicate. The diluted samples were added to confluent monolayers of Vero E6 cells and incubated for 1 h at 37°C in 5% carbon dioxide with rocking every 10 ± 5 min. The monolayers were then overlaid with a 1:1 mix of 2.5% Avicel (FMC Biopolymer, Philadelphia, PA, USA), diluted in 1 mL of 2X Eagle’s Minimum Essential Medium (Thermo Fisher) containing 4% heat-inactivated FBS, and incubated at 37°C for 3 d. After incubation, the overlay was removed, and the cells were fixed and stained for 30 min with 0.2% (f/c) of gentian violet (Ricca Chemical, Arlington, TX, USA) in 10% neutral buffered formalin. After 30 min, the fixative/stain was removed, the plates were washed in water, and the plaques were counted.

### Immunology

#### Serum antibody testing (ELISA and neutralization assay).

Humoral immune response against NiV was evaluated by detecting IgM and IgG antibodies from serum collected on the days outlined in the study overview or at unscheduled euthanasia. In-house ELISAs were used to quantify NiV-specific IgM and IgG antibodies in serum samples. The IgG and IgM ELISA were performed as previously described [23,46]. An indirect IgG ELISA was performed using irradiated NiV-infected cell lysate as the coating antigen. Briefly, 96-well plates (Thermo Fisher Scientific) were coated with 1X ELISA coating buffer (5X ELISA coating buffer, BioLegend), 0.05 μg per well of antigen was added, and plates were incubated overnight at 4°C. Plates were washed with 1X phosphate-buffered saline (PBS; Thermo Fisher Scientific) plus 0.1% (v/v) TWEEN20 (Sigma-Aldrich) (PBST), blocked with 3% normal chicken serum (Abcam, Waltham, MA, USA) plus 2% milk (made from skim milk powder [Thermo Fisher] plus PBST), and serially diluted (blocking solution with 1:50 normal monkey serum). Heat-inactivated serum samples were added and incubated overnight at 4°C. Plates were washed, rabbit anti-monkey IgG (whole molecule) with horseradish peroxidase (Sigma) was added, and then 3,3’,5,5’-tetramethylbenzidine (TMB) substrate (Thermo Fisher) was added. The reaction was stopped using a stop solution for TMB substrates (Thermo Fisher), and absorbance was measured at 450 nm using a Tecan plate reader. The assay was performed in triplicate and included both positive and negative control sera.

IgM capture ELISAs were performed using isotype-specific capture antibodies coated on 96-well plates (0.2 μg per well) and incubated overnight at 4°C. After washing and blocking, heat-inactivated serum samples were serially diluted and added in triplicate, followed by overnight incubation at 4°C. The next day, NiV-infected Vero E6 cell lysate, previously inactivated by irradiation and processed by sonication, was added as antigen. Antigen binding was then detected using a rabbit anti-NiV-G antibody, followed by horseradish-peroxidase-conjugated goat anti-rabbit IgG (Sigma) and TMB substrate. Reactions were stopped, and absorbance was read at 450 nm. Each plate included positive and negative control sera. Data were analyzed accordingly.

Neutralizing antibody titers were measured using a plaque-reduction neutralization assay based on tissue culture infectious dose. Serial dilutions of sera were incubated with NiV and applied to Vero E6 cell monolayers. After 5 d, cytopathic effect was assessed by staining, and the neutralization titer was defined as the highest serum dilution yielding ≥50% protection.

#### Whole blood immune cell phenotyping by flow cytometry.

Whole blood was stained for flow cytometry assay following the protocol described earlier [47–49]. In summary, 100 µL of whole blood samples from EDTA tubes were incubated with Fc receptors (BioLegend, San Diego, CA, USA) for 10 min, followed by the addition of a surface antibody cocktail, which was comprised of a mixture of conjugated primary antibodies, including amine-reactive dye (Live Dead Aqua; Thermo Fisher), CD3 Alexa Fluor 700 (clone SP34–2; BD Biosciences, San Jose, CA, USA), CD4 BV421 (clone L200; BD Biosciences), CD8a BV650 (clone SK1; BioLegend), CD14 Pacific Blue (clone M5E2; BioLegend), CD20 BV570 (clone 2H7; BioLegend), and CD45 PE-CF594 (clone D058-1283; BD Biosciences), for 20 min more on ice in the dark. Red blood cells were lysed with FACSLyse buffer (BD Biosciences), washed with 2% heat-inactivated FBS (Sigma-Aldrich) + PBS (Gibco, Grand Island, NY, USA) + 2 mM EDTA (Invitrogen, Carlsbad, CA, USA) (PBS-2), and the cells were fixed for at least 30 min with a minimum volume of 500 μL BD Cytofix/Cytoperm buffer (BD Biosciences). After staining, fully inactivated cells were washed with 1X perm wash and resuspended in PBS; cells were assessed on a BD LSRII Fortessa cytometer. At least 50,000 events were acquired from each sample, and the data were analyzed using FlowJo software (version 10.10, FlowJo LLC, Ashland, OR, USA), with gating determined by fluorescence-minus-one controls. The absolute quantification of various cell populations was determined utilizing CBC data in conjunction with flow cytometry analysis.

#### Multiplex cytokine analysis in plasma.

Circulatory cytokine, chemokine, and growth factor responses were assessed using the MILLIPLEX MAP Non-Human Primate Cytokine Magnetic Bead Panel – Premixed 23 Plex (MilliporeSigma, St. Louis, MO, USA) on a Luminex MAGPIX Multiplexing System (Luminex Corporation, Austin, TX, USA). Analytes in this panel measured G-CSF, GM-CSF, IFNγ, IL-1β, IL-1RA, IL-2, IL-4, IL-5, IL-6, IL-8, IL-10, IL-12/23 (p40), IL-13, IL-15, IL-17A, IL-18, MCP-1, MIP-1β, MIP-1α, sCD40L, TGF-α, TNF-α, and VEGF. Plasma samples were isolated from whole blood samples and stored at -80°C until examined. Samples were run in duplicate and processed according to the manufacturer’s instructions. All reagents were equilibrated to room temperature, and premixed beads were resuspended by vortexing prior to use. Standard curves were prepared by serial four-fold dilution of reconstituted standards in assay buffer, with assay buffer alone used as a blank.

The assay plates were pre-washed and subsequently loaded with either a serum matrix for controls and standards or an assay buffer for test samples. This was followed by the addition of 25 µL of plasma or control material, which had been thawed, vortexed, and centrifuged, along with 25 µL of resuspended beads. Plates were sealed and incubated overnight at 4°C with shaking. The following day, plates were washed twice using a handheld magnetic separator and incubated for 1 h at room temperature with the detection antibody. Following a 30-minute incubation period with Streptavidin-PE, the plates underwent an additional washing step. Subsequently, sheath fluid was introduced, and data acquisition was performed by loading the samples into the MAGPIX instrument for subsequent reading and analysis.

Raw data were processed using Bio-Results Generator 3.0 and Bio-Plex Manager software using a 5-parameter logistic or spine curve-fitting method as done previously [48]. Minimum detectable concentrations for each cytokine were calculated and included in the graphical output. Final visualization and analysis were performed using GraphPad Prism. Baseline samples collected on separate pre-exposure days were combined into a single “B1/B2” group for analysis.

### Imaging

The imaging schedule is described in the study design provided in Fig 1. The imaging methods used have been used in numerous studies in the past and have been previously published [22]; They are recapped in brief below:

#### MRI.

Following CT imaging, the AGMs were transported to the MRI suite. An intravenous catheter was inserted into the front leg vein of each animal before entering the imaging room. This catheter was connected to a line prefilled with gadobutrol for manual injection, followed by a saline bolus to flush the system. Structural, quantitative, and functional brain MR images were acquired using a pediatric head/supine coil with a head element section containing four channels on 3T Philips Achieva MRI scanner.

A series of imaging sequences was conducted to assess the brain for signs of inflammation or lesions potentially resulting from NiV infection or its downstream consequences. These sequences included T2-weighted turbo spin-echo, T1-weighted fast-field-echo (FFE), diffusion tensor (DT), and T2-weighted FLAIR. T1-weighted FFE and T2-weighted FLAIR images were acquired both before and after contrast enhancement. T1 maps were generated using a dual flip angle method from T1-weighted FFE images. Diffusion-weighted images, trace of the DT, and fractional anisotropy maps were constructed from DT images. Most MR images were obtained in the transaxial plane.

#### CT.

CT was utilized for real-time assessment of disease progression, primarily in the lungs, and to verify consistency with other clinical parameters. Baseline CT scans were conducted before inoculation, followed by scans on several post-exposure days until the conclusion of the study, as outlined in the study design.

High-resolution whole-body CT scans were conducted in the transaxial plane on a Philips Gemini Time of Flight PET CT scanner specifically designed to function in a BSL-4 environment (Philips USA). The CT scans were acquired using the following parameters: a tube voltage of 140 kilovoltage peak (kVp) and a tube current of 300 milliampere-seconds (mAs) per section. The slice thickness was set at 1 mm, with a slice increment of 0.5 mm. The pitch was configured at 0.438 mm, and the collimation was set at 16 × 0.75 mm. Each rotation of the scanner was completed in 0.5 s. Images were reconstructed with a pixel size of 0.5 mm within a 250-mm field of view. The section spacing was also set to 0.5 mm. A breath-hold was employed to obtain motion-free images.

#### Qualitative image analysis.

All images were reviewed by image analysts with several years of experience evaluating CT and MR images, as well as by board-certified clinical radiologists.

***MRI*:** MR imaging sequences can identify cytotoxic and vasogenic edema as hyperintense regions on T2-weighted and FLAIR images, changes in diffusion patterns (such as restricted diffusion by hypointense signals on trace DT maps), and blood–brain barrier disruption by comparing T1 maps computed from T1-weighted FFE images before and after contrast injection. Encephalitic or other inflammatory changes can also be captured by changes in the images by comparison with baseline data.

***CT*:** Lung CTs were used to evaluate abnormalities such as bronchial wall thickening, peribronchovascular infiltrates, consolidations, ground-glass opacities, air bronchograms, and any overlapping areas of the aforementioned conditions.

#### Quantitative image analysis.

***CT*:** For each CT scan, the lungs were segmented using a deep learning workflow implemented within MIM Software, as described in previous work [50]. A histogram-based analysis was then conducted on the segmented lung area. The PCLH was calculated as previously described by Solomon et al. (2014) [51]. In summary, we first analyzed the image histogram of the baseline pre-inoculation scan for each animal. The 5% cutoff was identified as the value below which 95% of voxels had the lowest Hounsfield unit (HU) values. As the disease progressed, more voxels exhibited higher HU values due to consolidation and infiltrates replacing normal aerated lung tissues. These changes are represented on the histogram by an increase in voxels with intensities above the 5% cutoff value. We then calculated the PCLH from baseline using the formula [(*V*n - *V*b)/*V*b] × 100, where *V*b is baseline volume and *V*n is volume at time n. This PCLH by volume was tracked for all animals.

### Histopathology and molecular pathology

Necropsies were performed by a board-certified veterinary pathologist, and the following representative tissues were collected for histopathological and molecular pathological analyses: skin, nares, peripheral lymph nodes, spleen, adrenal gland, kidneys, bone marrow, thymus, heart, trachea, esophagus, thyroid gland, parathyroid gland, lungs, liver, gastrointestinal tract, pancreas, gonads, skeletal muscle, eyes, brain (cerebrum, cerebellum, brainstem), and pituitary gland. Additional representative tissues were collected to include any lesions identified on gross necropsy, CT, or MRI. Tissue samples were fixed by immersion in 10% neutral buffered formalin for at least 72 h prior to removal from the biocontainment suite.

Fixed tissues were routinely trimmed, processed, and embedded in paraffin, sectioned at 5–6 µm thickness, and mounted on glass slides. Sections were then stained with hematoxylin and eosin and cover-slipped for microscopic evaluation. Slides prepared for immunohistochemistry were stained using primary polyclonal rabbit antibodies against NiV glycoprotein, secondary anti-rabbit antibodies complexed with a 3–3’ diaminobenzidene chromogen system (Biocare Medical, Pacheco, CA, USA) and counterstained with hematoxylin. *In situ* hybridization (ISH) was performed using the RNAscope 2.5 HD RED kit (Advanced Cell Diagnostics, Newark, CA, USA) to detect NiV genomic RNA in formalin-fixed, paraffin-embedded tissues, according to the manufacturer’s instructions. Briefly, 20 ZZ probe pairs targeting the NiV N gene were designed and synthesized by Advanced Cell Diagnostics. Signal hybridization was visualized with red chromogen and hematoxylin counterstain. All slides were analyzed and interpreted by a board-certified veterinary pathologist using an Olympus BX-46 light microscope.

### Statistical analysis

Formal statistical comparisons were not performed for this study because animals were euthanized at different time points, resulting in datasets that are not directly comparable across groups. In addition, the number of animals within each outcome group and time point was too small to support meaningful or reliable statistical testing. Therefore, all data are presented descriptively to illustrate overall trends and patterns associated with disease progression and outcome.

## Supporting information

S1 Fig Serial brain MRI scans for animals exposed to NiV-M and interpretation by a radiologist. (A) Longitudinal brain MRI scans over time of the cohort 1 individual animals exposed to NiV-M. (B) Longitudinal brain MRI scans over time of the cohort 2 individual animals exposed to NiV-M.
(TIF)

S2 Fig Serial brain MRI scans for animals exposed to NiV-B and interpretation by a radiologist. (A) Longitudinal brain MRI scans over time of the cohort 1 individual animals exposed to NiV-B. (B) Longitudinal brain MRI scans over time of the cohort 2 individual animals exposed to NiV-B.
(TIF)

S3 Fig Serial chest CT scans for animals exposed to NiV-M and interpretation by a radiologist. (A) Longitudinal chest CT scans over time of the cohort 1 individual animals exposed to NiV-M. (B) Longitudinal chest CT scans over time of the cohort 2 individual animals exposed to NiV-M.
(TIF)

S4 Fig Serial chest CT scans for animals exposed to NiV-B and interpretation by a radiologist. (A) Longitudinal chest CT scans over time of the cohort 1 individual animals exposed to NiV-B. (B) Longitudinal chest CT scans over time of the cohort 2 individual animals exposed to NiV-B.
(TIF)

S5 Fig Raw Data of Individual Animals by Outcome Groups (weights, temps, CBC, chemistry, RT-qPCR values in blood, serum, swabs, tissues, viral titers of tissues). (TIF)

S6 Fig Raw Data of Individual Animals by Outcome Groups (weights, temps, CBC, chemistry, RT-qPCR values in blood, serum, swabs, tissues, viral titers of tissues). (TIF)

S7 Fig Raw Data of Individual Animals by Outcome Groups (weights, temps, CBC, chemistry, RT-qPCR values in blood, serum, swabs, tissues, viral titers of tissues). (TIF)

S8 Fig Raw Data of Individual Animals by Outcome Groups (weights, temps, CBC, chemistry, RT-qPCR values in blood, serum, swabs, tissues, viral titers of tissues). (TIF)

S9 Fig Raw Data of Individual Animals by Outcome Groups (weights, temps, CBC, chemistry, RT-qPCR values in blood, serum, swabs, tissues, viral titers of tissues). (TIF)

S10 Fig Raw Data of Individual Animals by Outcome Groups (weights, temps, CBC, chemistry, RT-qPCR values in blood, serum, swabs, tissues, viral titers of tissues). (TIF)

S11 Fig Raw Data of Individual Animals by Outcome Groups (weights, temps, CBC, chemistry, RT-qPCR values in blood, serum, swabs, tissues, viral titers of tissues). (TIF)

S12 Fig Raw Data of Individual Animals by Outcome Groups (weights, temps, CBC, chemistry, RT-qPCR values in blood, serum, swabs, tissues, viral titers of tissues). (TIF)

S13 Fig Raw Data of Individual Animals by Outcome Groups (weights, temps, CBC, chemistry, RT-qPCR values in blood, serum, swabs, tissues, viral titers of tissues). (TIF)

S14 Fig Raw Data of Individual Animals by Outcome Groups (weights, temps, CBC, chemistry, RT-qPCR values in blood, serum, swabs, tissues, viral titers of tissues). (TIF)

S15 Fig Raw Data of Individual Animals by Outcome Groups (weights, temps, CBC, chemistry, RT-qPCR values in blood, serum, swabs, tissues, viral titers of tissues). (TIF)

S16 Fig Raw Data of Individual Animals by Outcome Groups (weights, temps, CBC, chemistry, RT-qPCR values in blood, serum, swabs, tissues, viral titers of tissues). (TIF)

S17 Fig Raw Data of Individual Animals by Outcome Groups (weights, temps, CBC, chemistry, RT-qPCR values in blood, serum, swabs, tissues, viral titers of tissues). (TIF)

S18 Fig Raw Data of Individual Animals by Outcome Groups (weights, temps, CBC, chemistry, RT-qPCR values in blood, serum, swabs, tissues, viral titers of tissues). (TIF)

S19 Fig Raw Data of Individual Animals by Outcome Groups (weights, temps, CBC, chemistry, RT-qPCR values in blood, serum, swabs, tissues, viral titers of tissues). (TIF)

S20 Fig Raw Data of Individual Animals by Outcome Groups (weights, temps, CBC, chemistry, RT-qPCR values in blood, serum, swabs, tissues, viral titers of tissues). (TIF)

S21 Fig Raw Data of Individual Animals by Outcome Groups (weights, temps, CBC, chemistry, RT-qPCR values in blood, serum, swabs, tissues, viral titers of tissues). (TIF)

S22 Fig Histologic Detection of NiV by Immunohistochemistry (IHC) and In Situ Hybridization (ISH) across Tissues. (TIF)
