# Supplementary figures and images for: Large-particle aerosol exposure to the Bangladesh or Malaysia strain of Nipah virus results in markedly divergent disease presentation in African Green Monkeys
Source: PLoS Pathog. 2025 Dec 29;21(12):e1013835. doi: 10.1371/journal.ppat.1013835 (PMC12768417; doi:10.1371/journal.ppat.1013835)

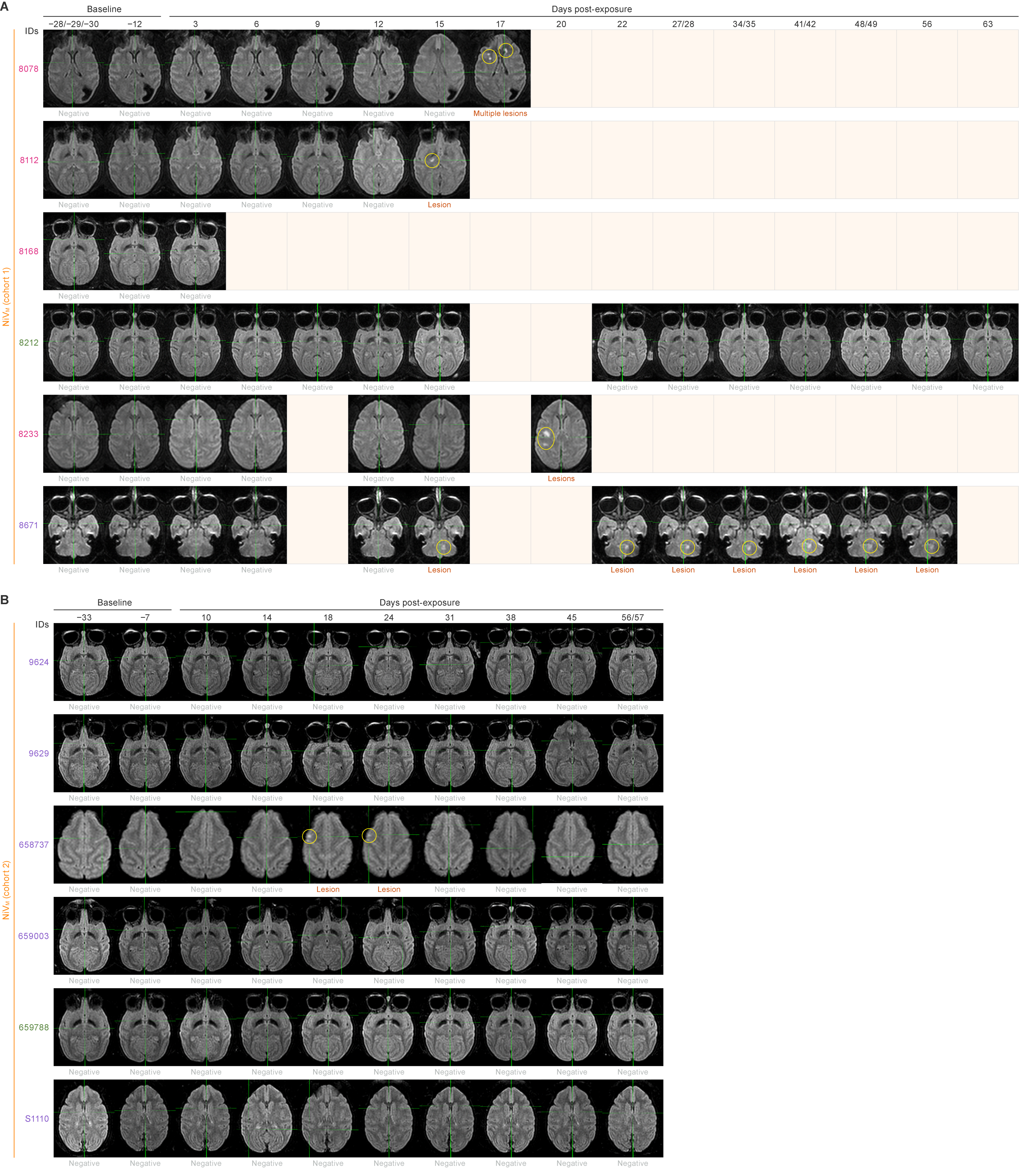

Supplement: S1 Fig — (A) Longitudinal brain MRI scans over time of the cohort 1 individual animals exposed to NiV-M. (B) Longitudinal brain MRI scans over time of the cohort 2 individual animals exposed to NiV-M. (TIF) [file ppat.1013835.s001.tif]

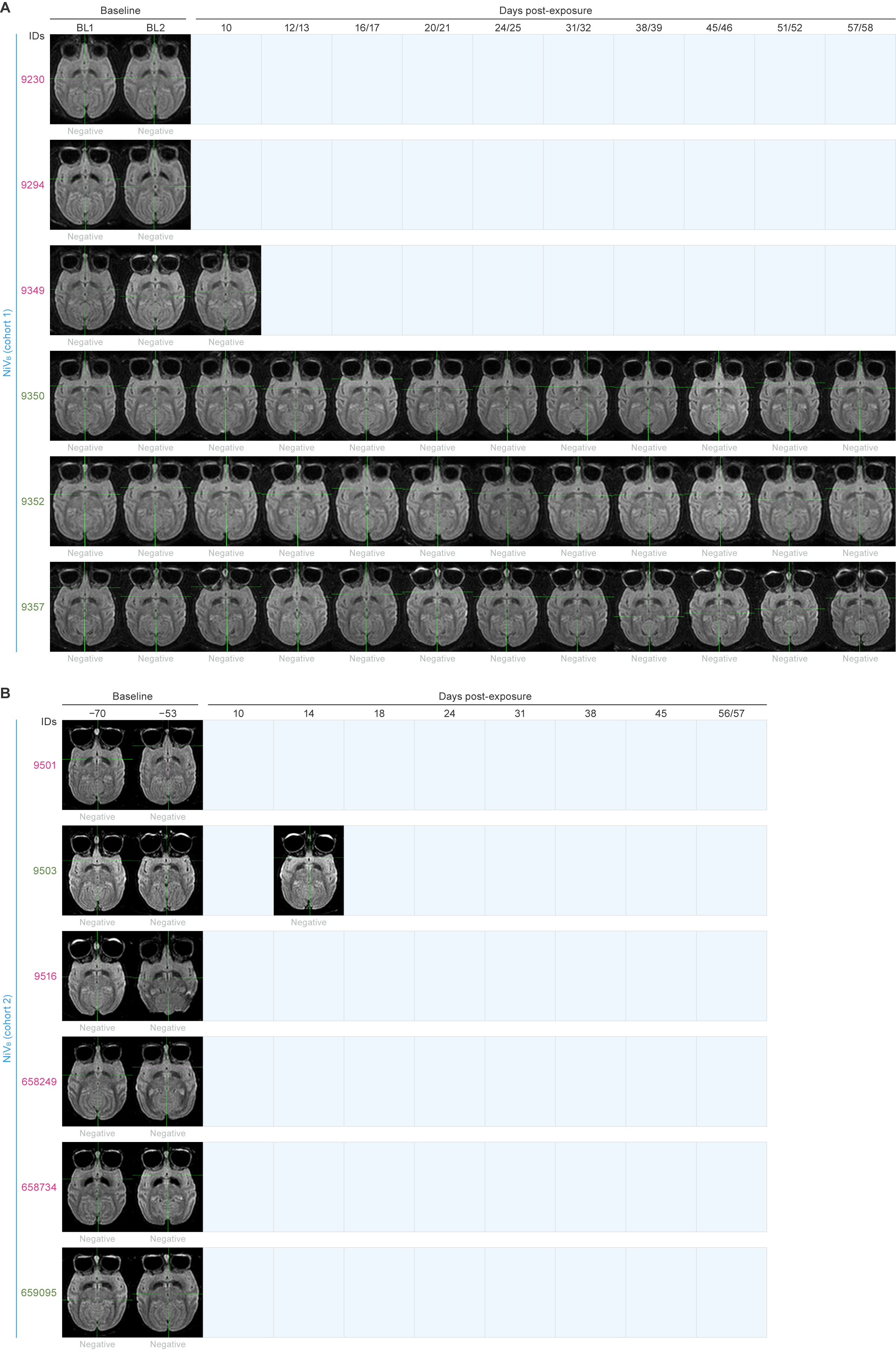

Supplement: S2 Fig — (A) Longitudinal brain MRI scans over time of the cohort 1 individual animals exposed to NiV-B. (B) Longitudinal brain MRI scans over time of the cohort 2 individual animals exposed to NiV-B. (TIF) [file ppat.1013835.s002.tif]

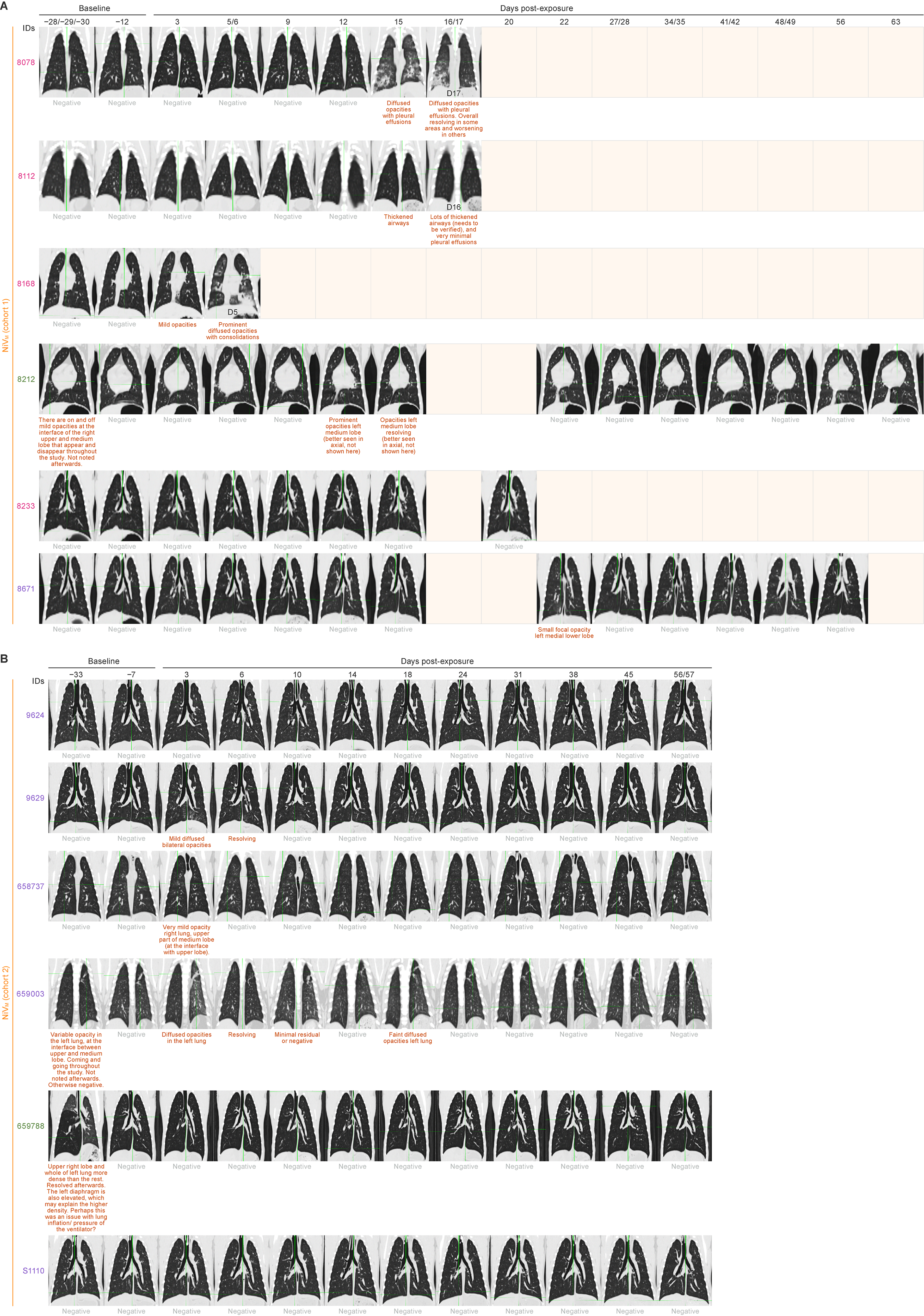

Supplement: S3 Fig — (A) Longitudinal chest CT scans over time of the cohort 1 individual animals exposed to NiV-M. (B) Longitudinal chest CT scans over time of the cohort 2 individual animals exposed to NiV-M. (TIF) [file ppat.1013835.s003.tif]

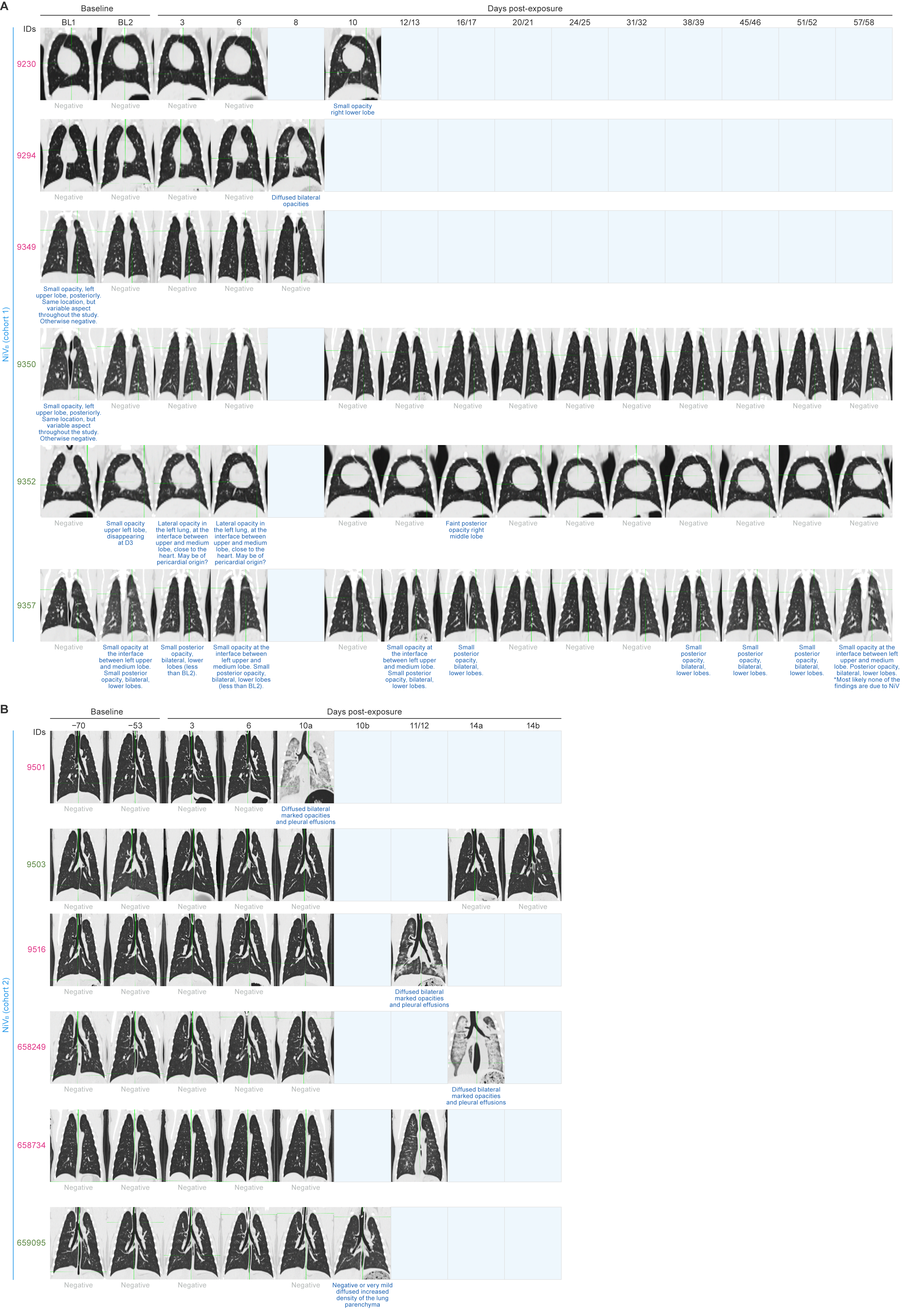

Supplement: S4 Fig — (A) Longitudinal chest CT scans over time of the cohort 1 individual animals exposed to NiV-B. (B) Longitudinal chest CT scans over time of the cohort 2 individual animals exposed to NiV-B. (TIF) [file ppat.1013835.s004.tif]

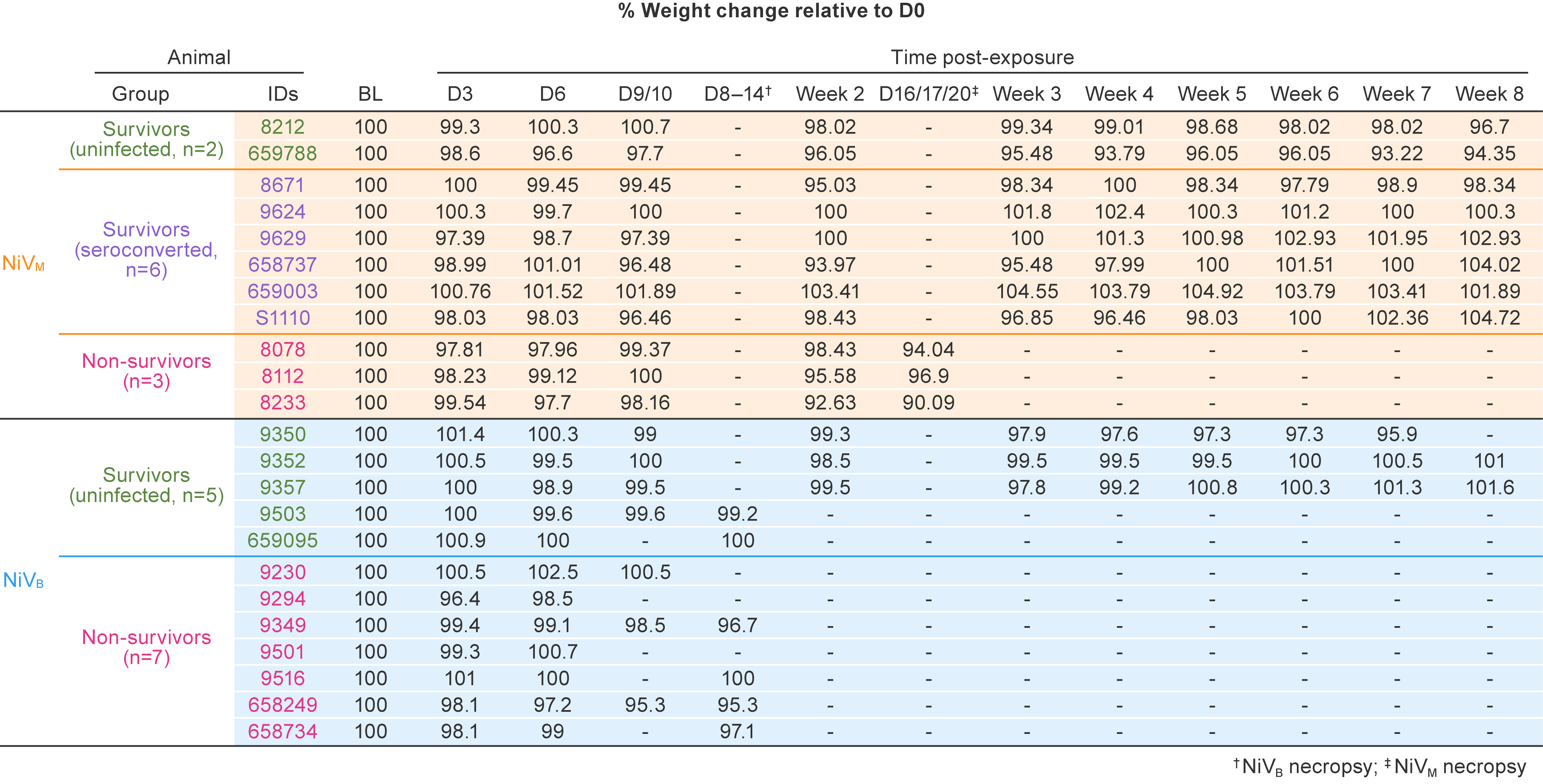

Supplement: S5 Fig — (TIF) [file ppat.1013835.s005.tif]

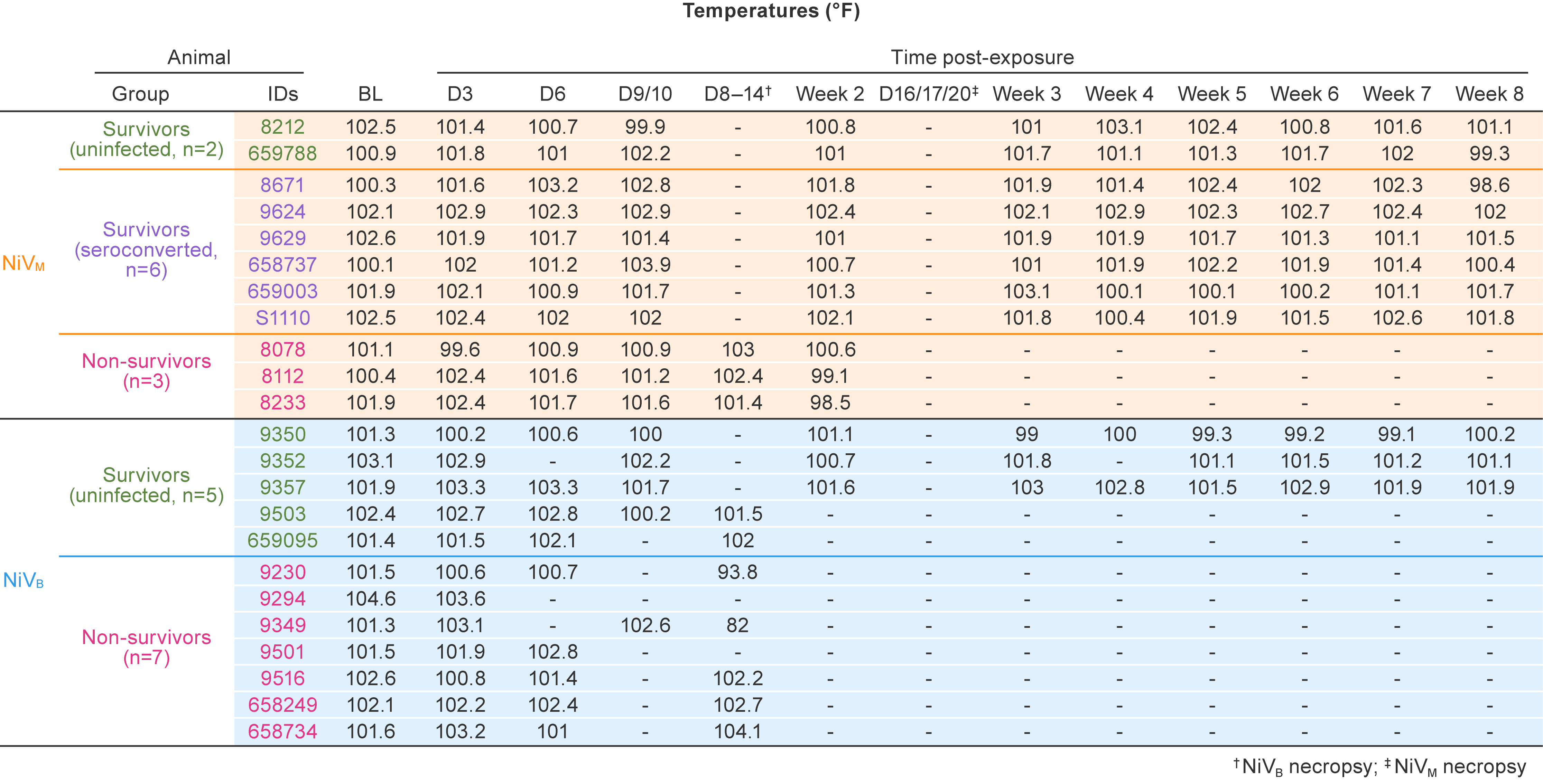

Supplement: S6 Fig — (TIF) [file ppat.1013835.s006.tif]

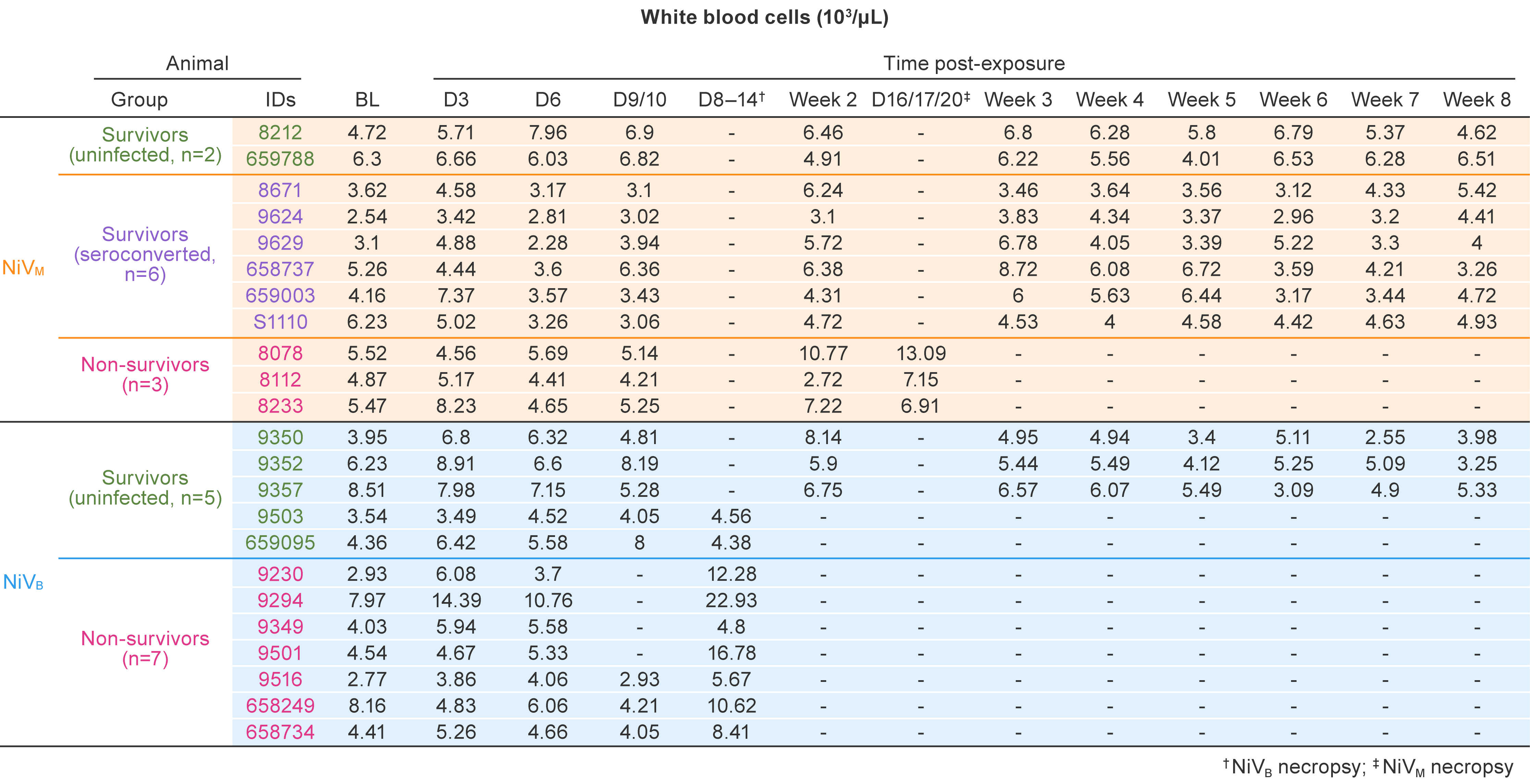

Supplement: S7 Fig — (TIF) [file ppat.1013835.s007.tif]

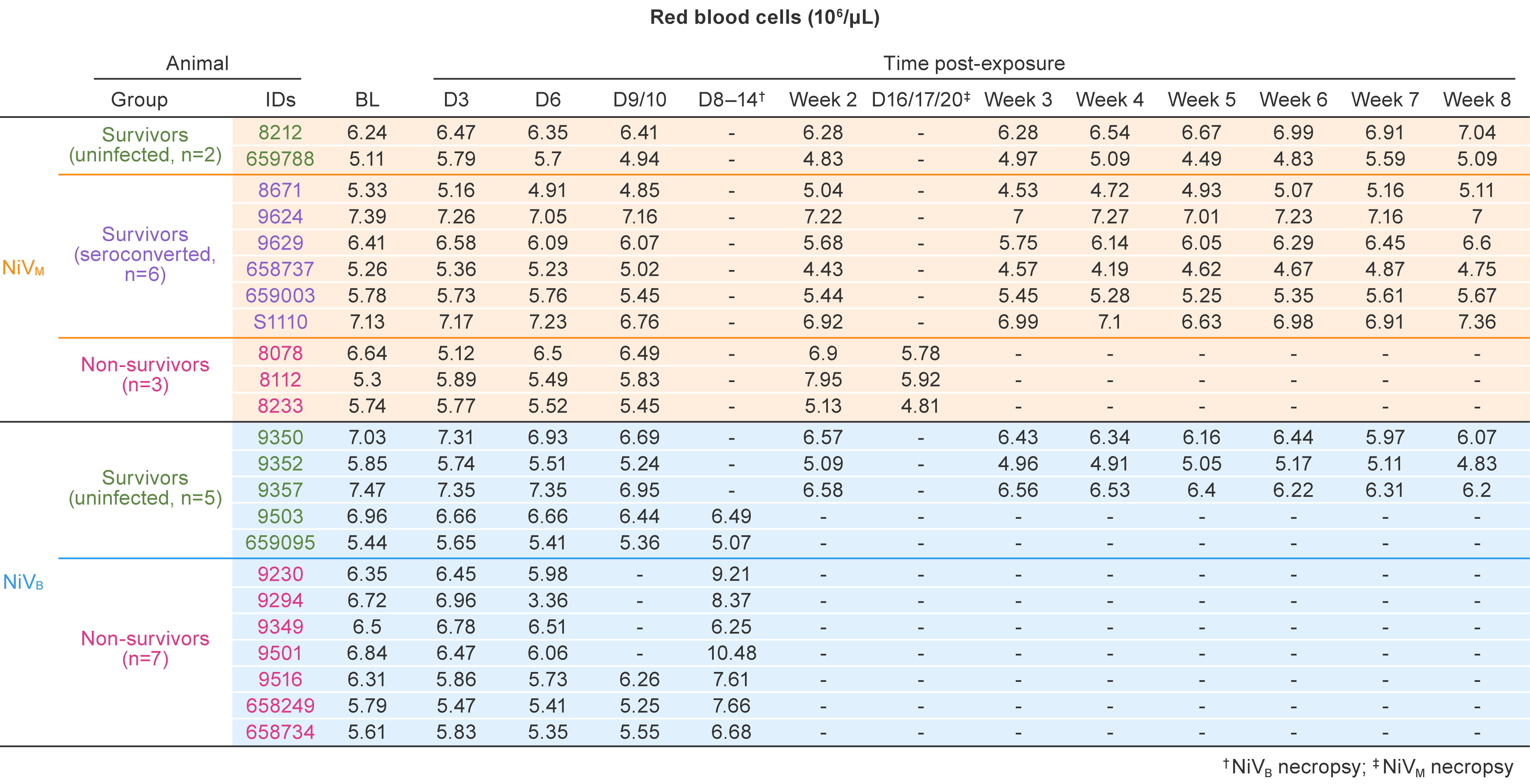

Supplement: S8 Fig — (TIF) [file ppat.1013835.s008.tif]

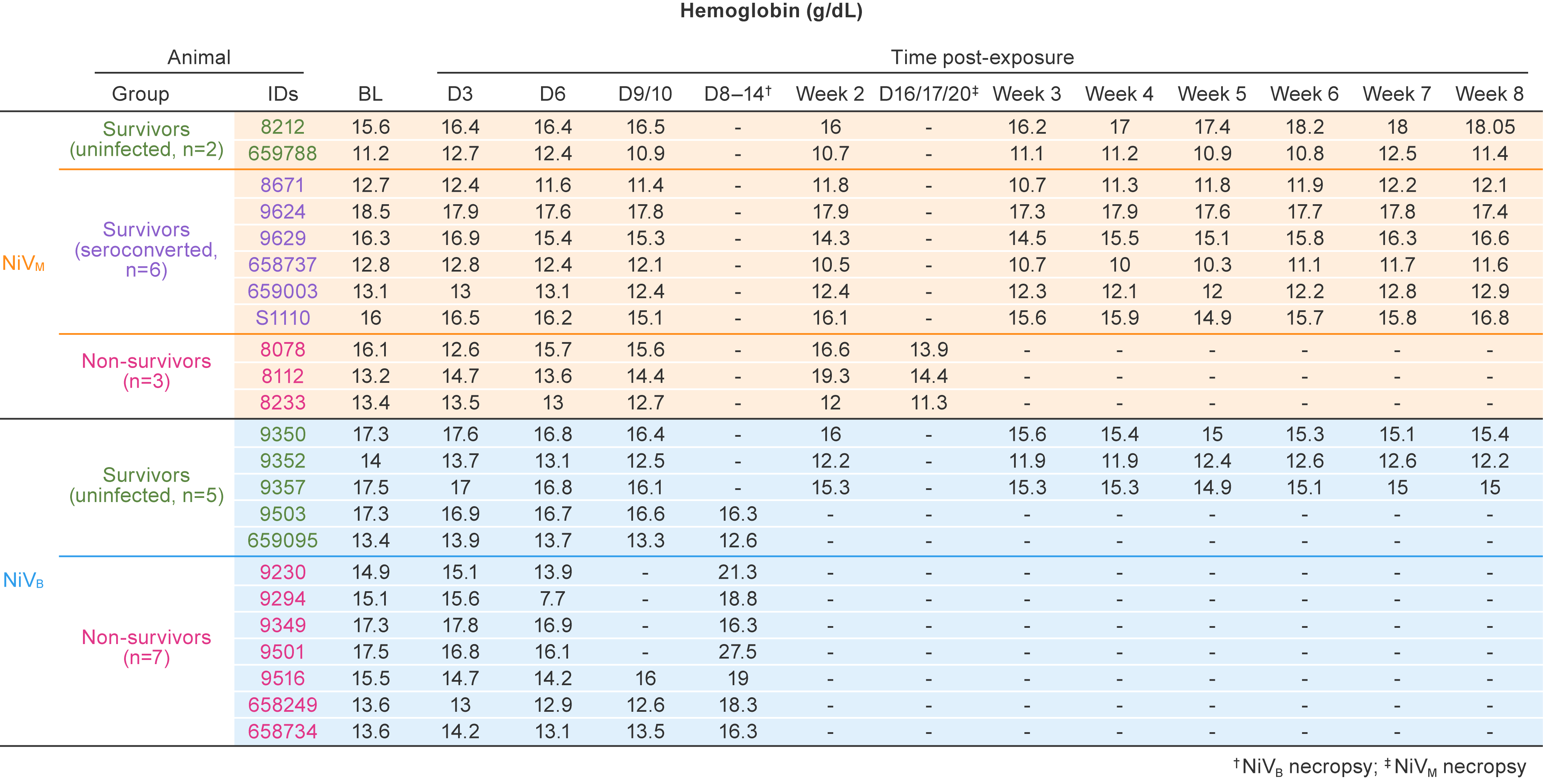

Supplement: S9 Fig — (TIF) [file ppat.1013835.s009.tif]

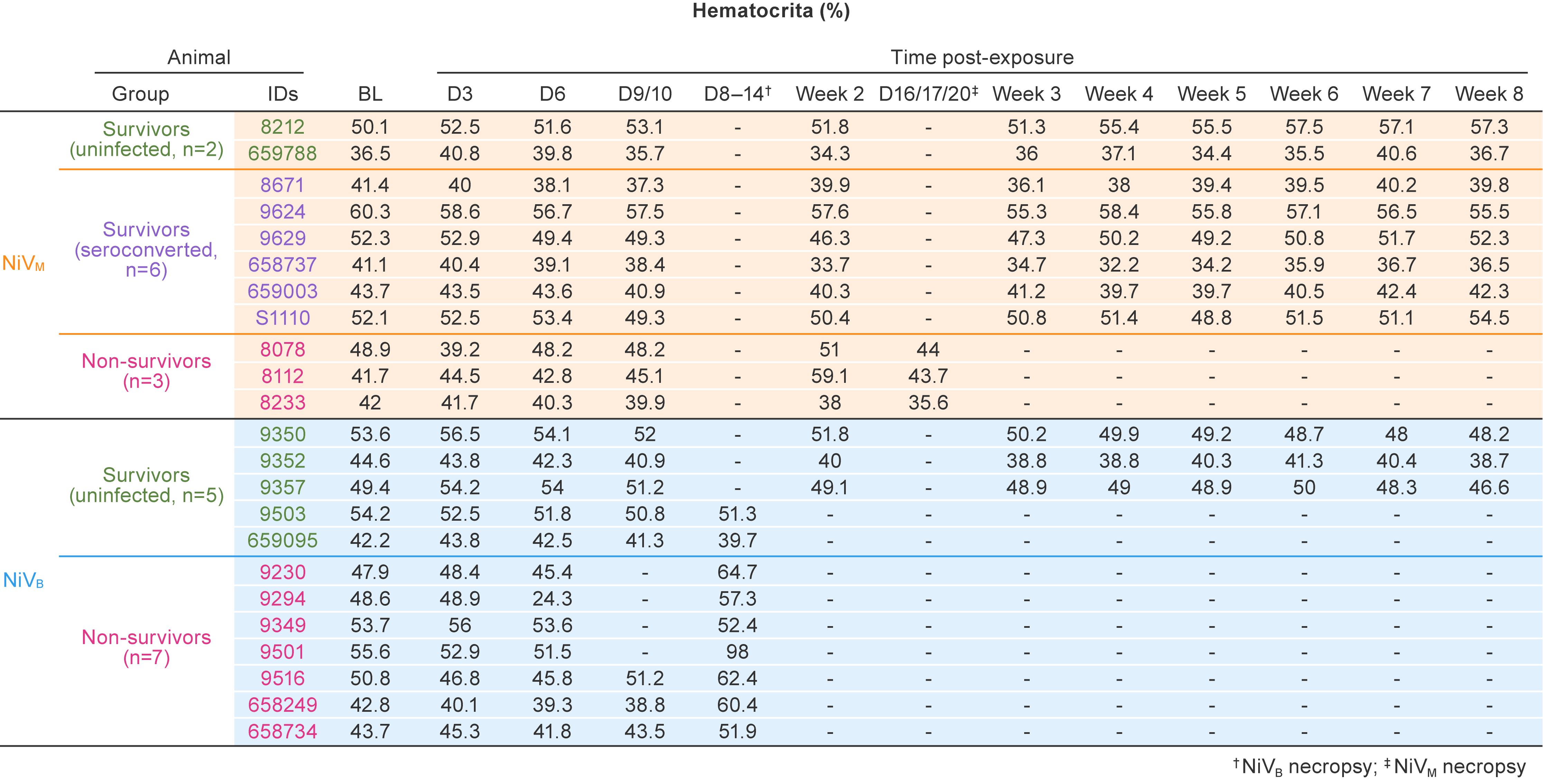

Supplement: S10 Fig — (TIF) [file ppat.1013835.s010.tif]

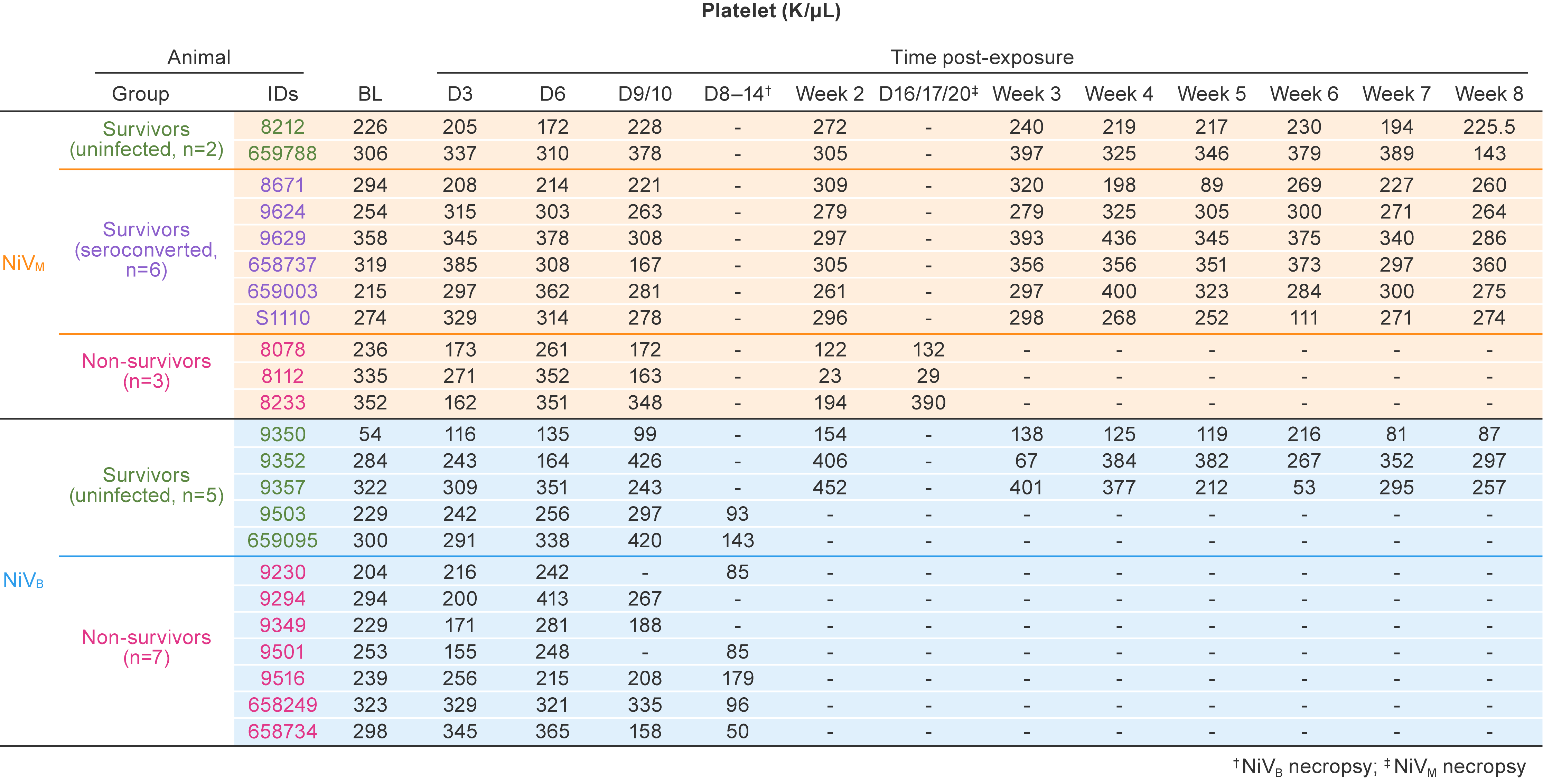

Supplement: S11 Fig — (TIF) [file ppat.1013835.s011.tif]

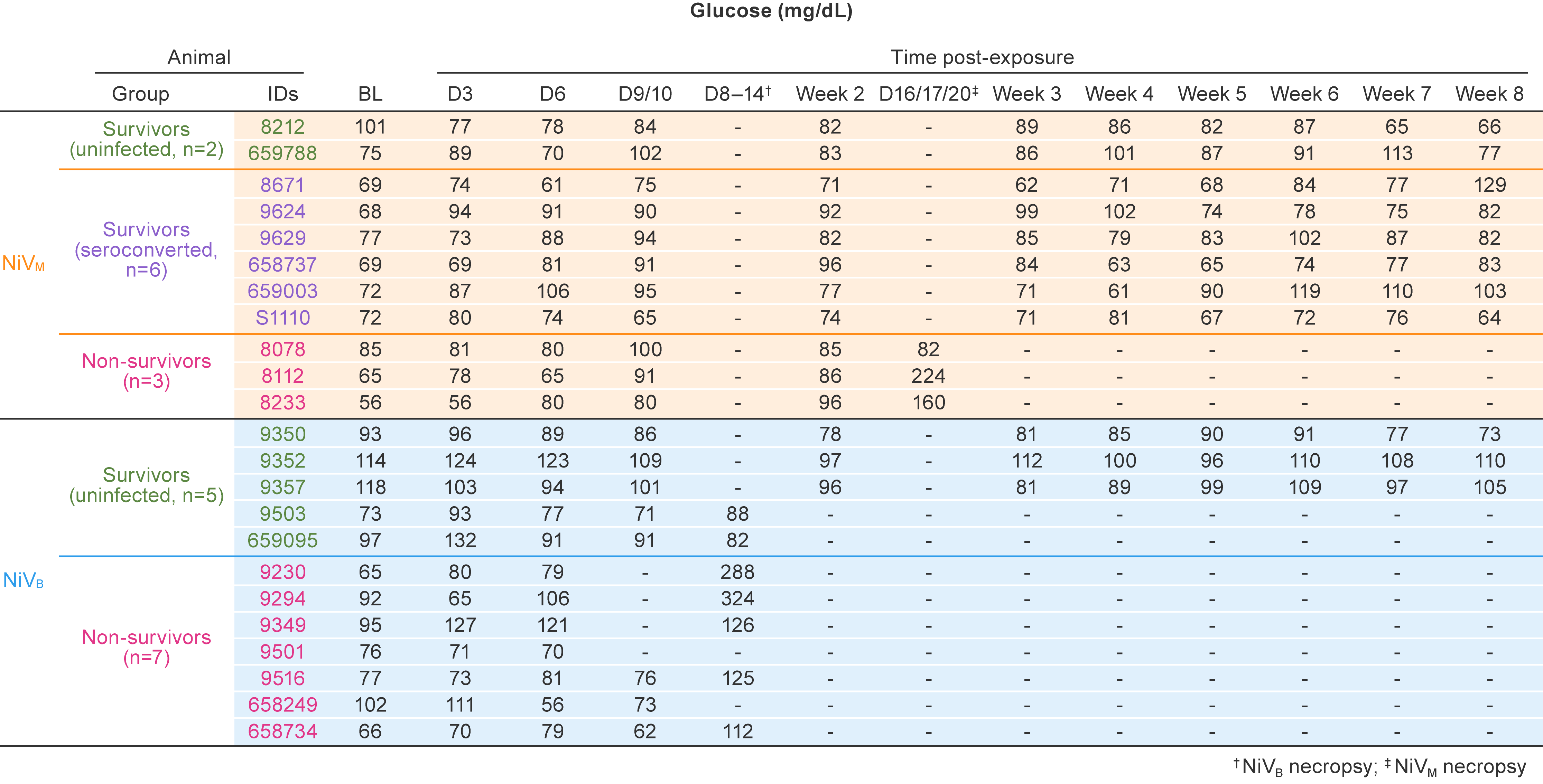

Supplement: S12 Fig — (TIF) [file ppat.1013835.s012.tif]

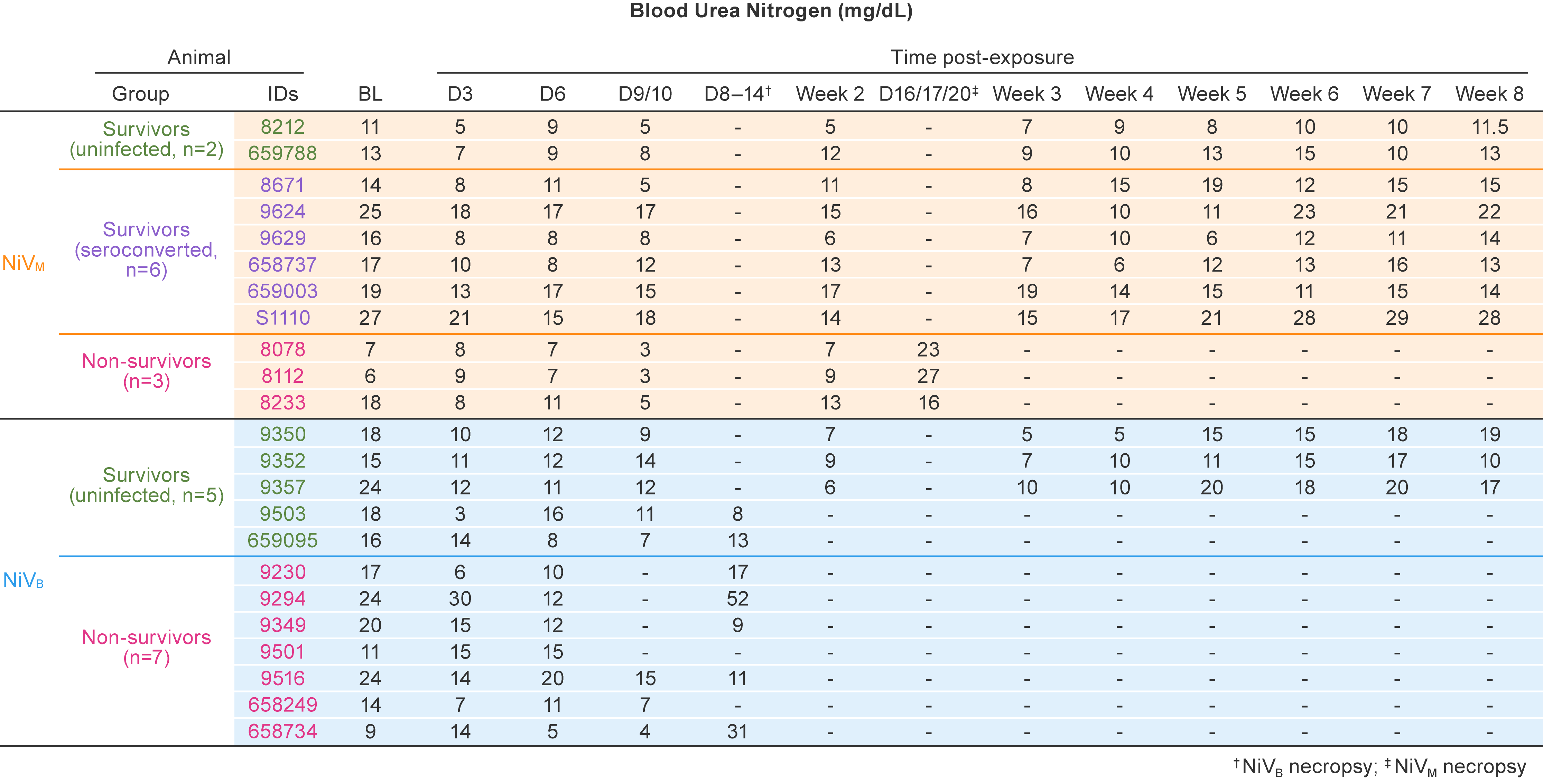

Supplement: S13 Fig — (TIF) [file ppat.1013835.s013.tif]

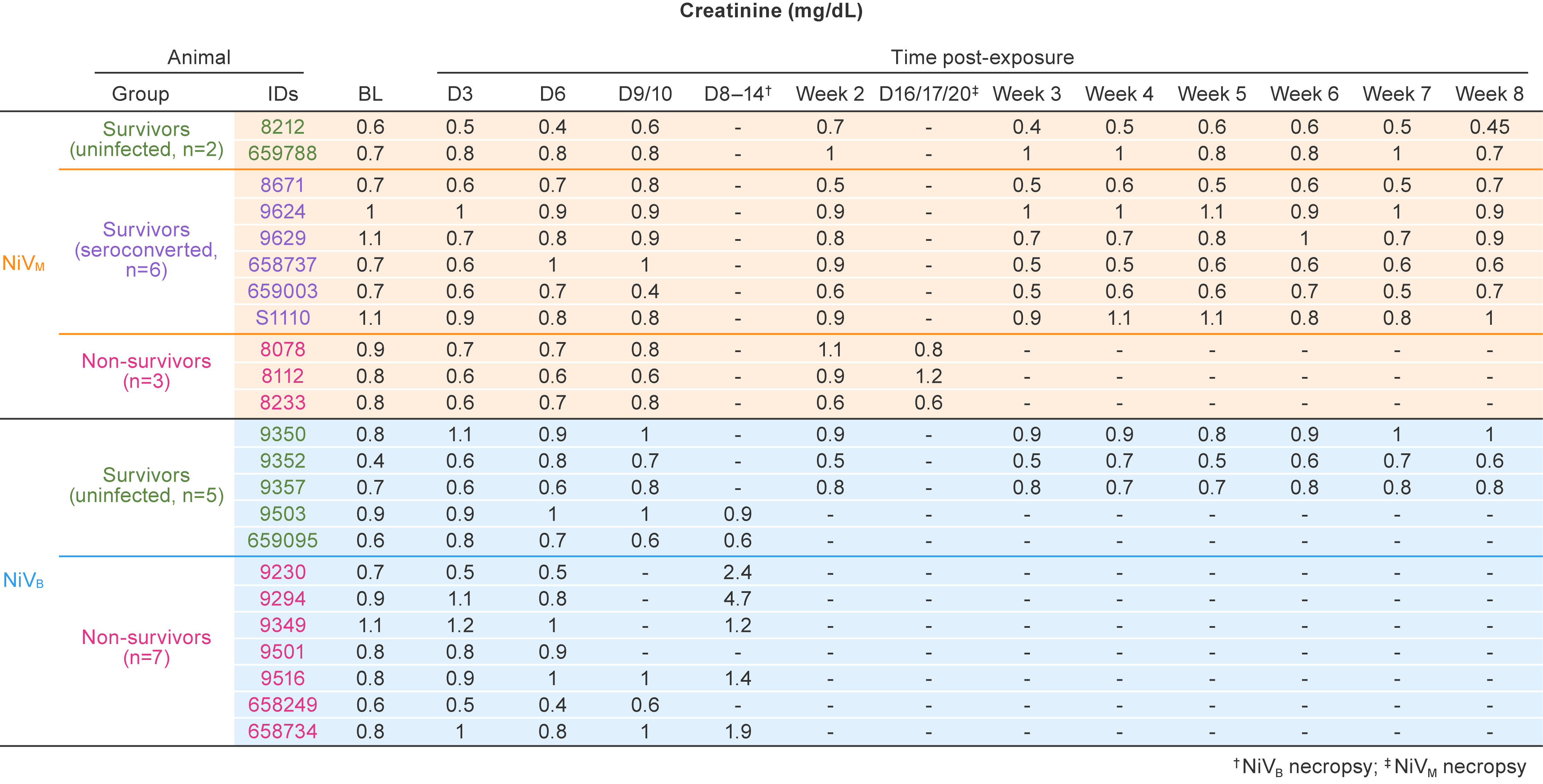

Supplement: S14 Fig — (TIF) [file ppat.1013835.s014.tif]

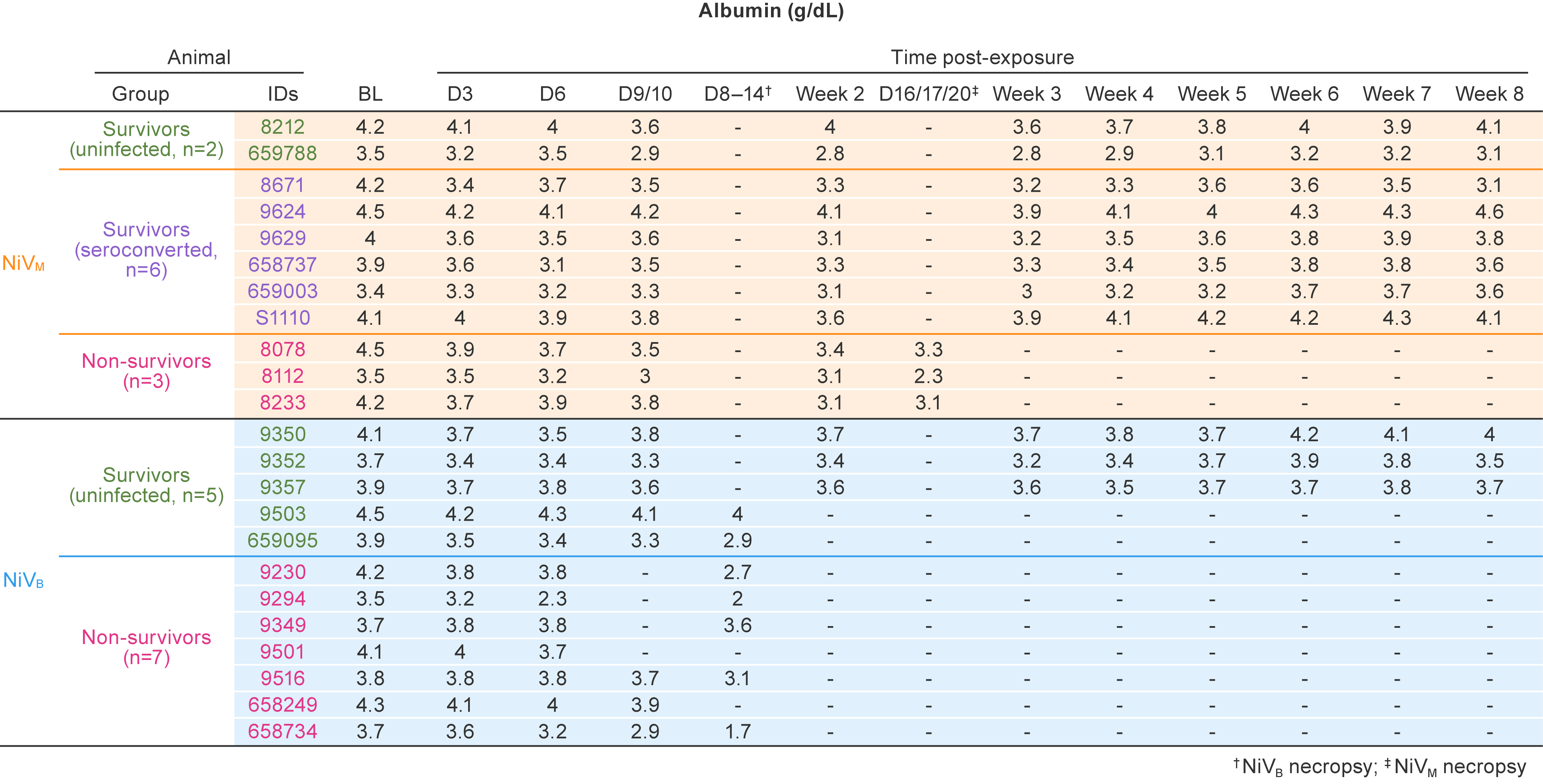

Supplement: S15 Fig — (TIF) [file ppat.1013835.s015.tif]

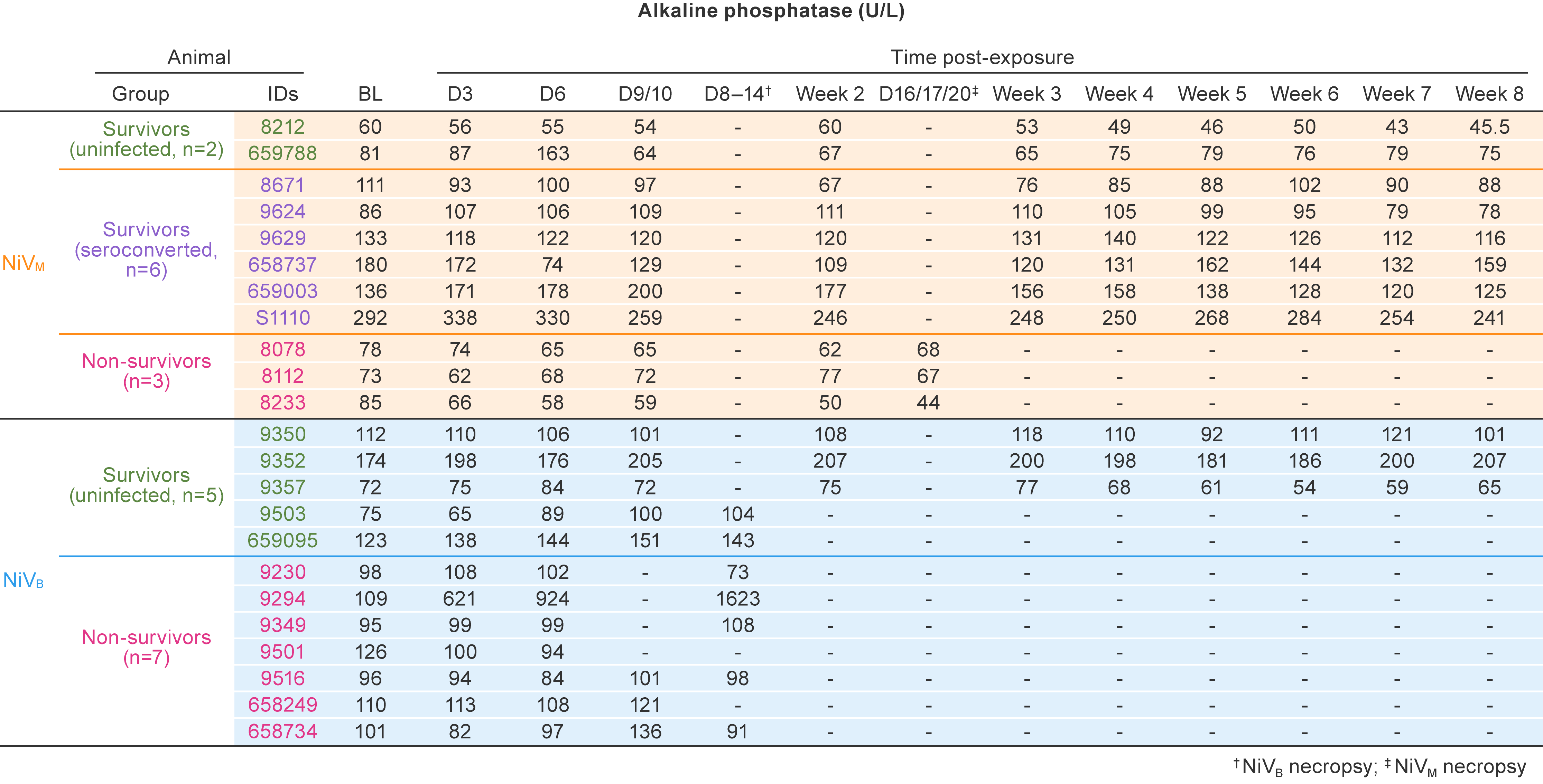

Supplement: S16 Fig — (TIF) [file ppat.1013835.s016.tif]

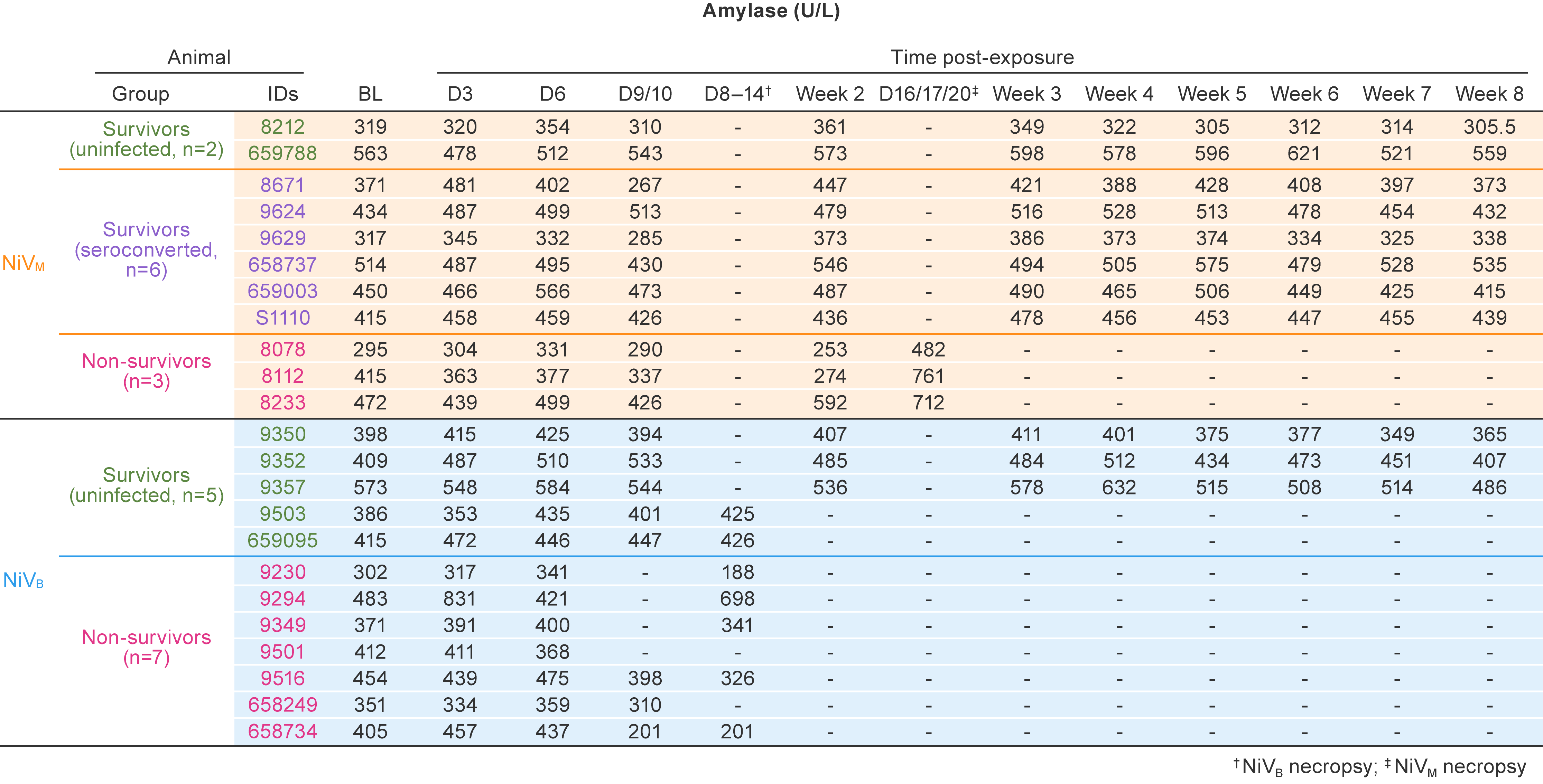

Supplement: S17 Fig — (TIF) [file ppat.1013835.s017.tif]

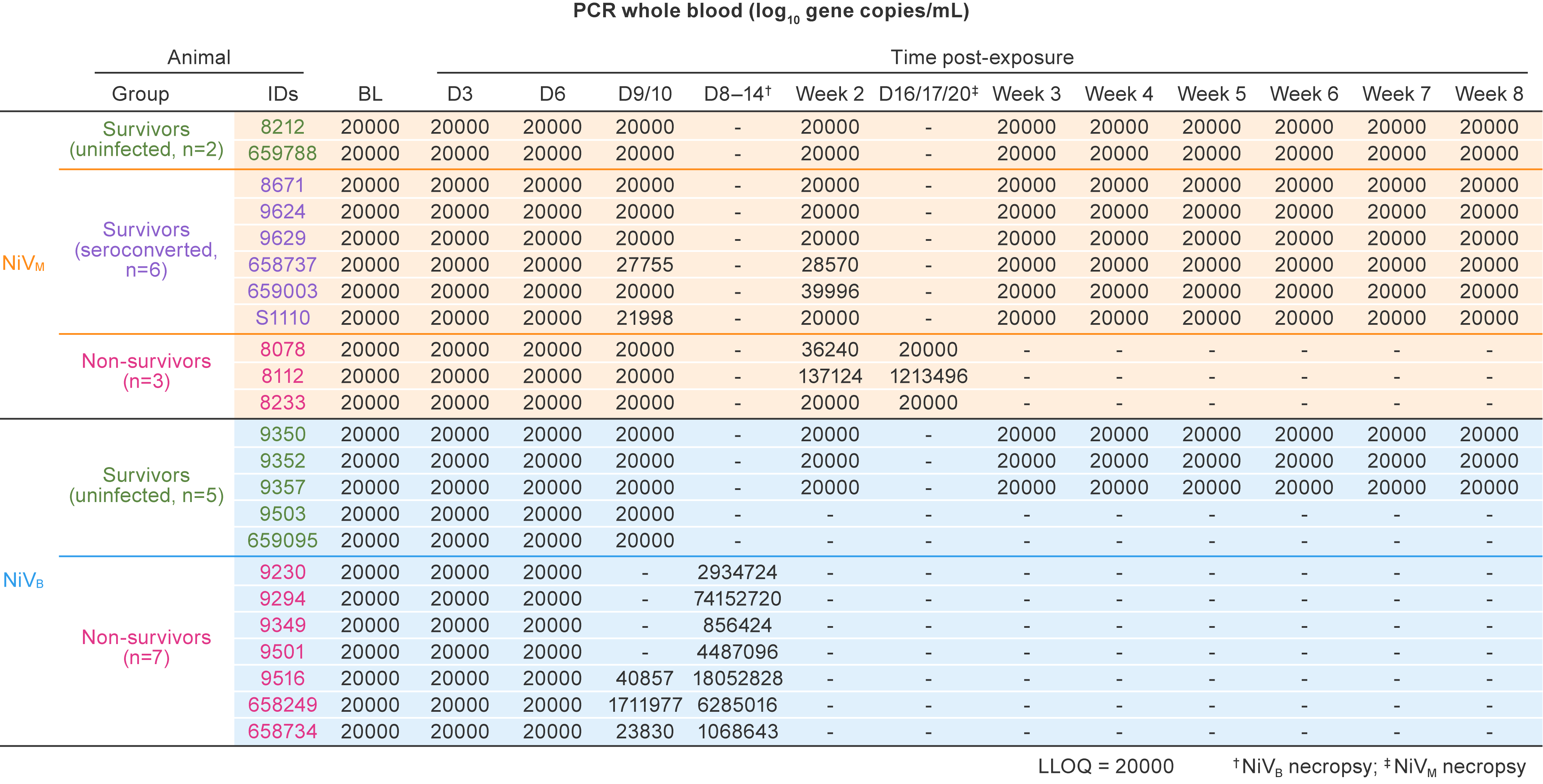

Supplement: S18 Fig — (TIF) [file ppat.1013835.s018.tif]

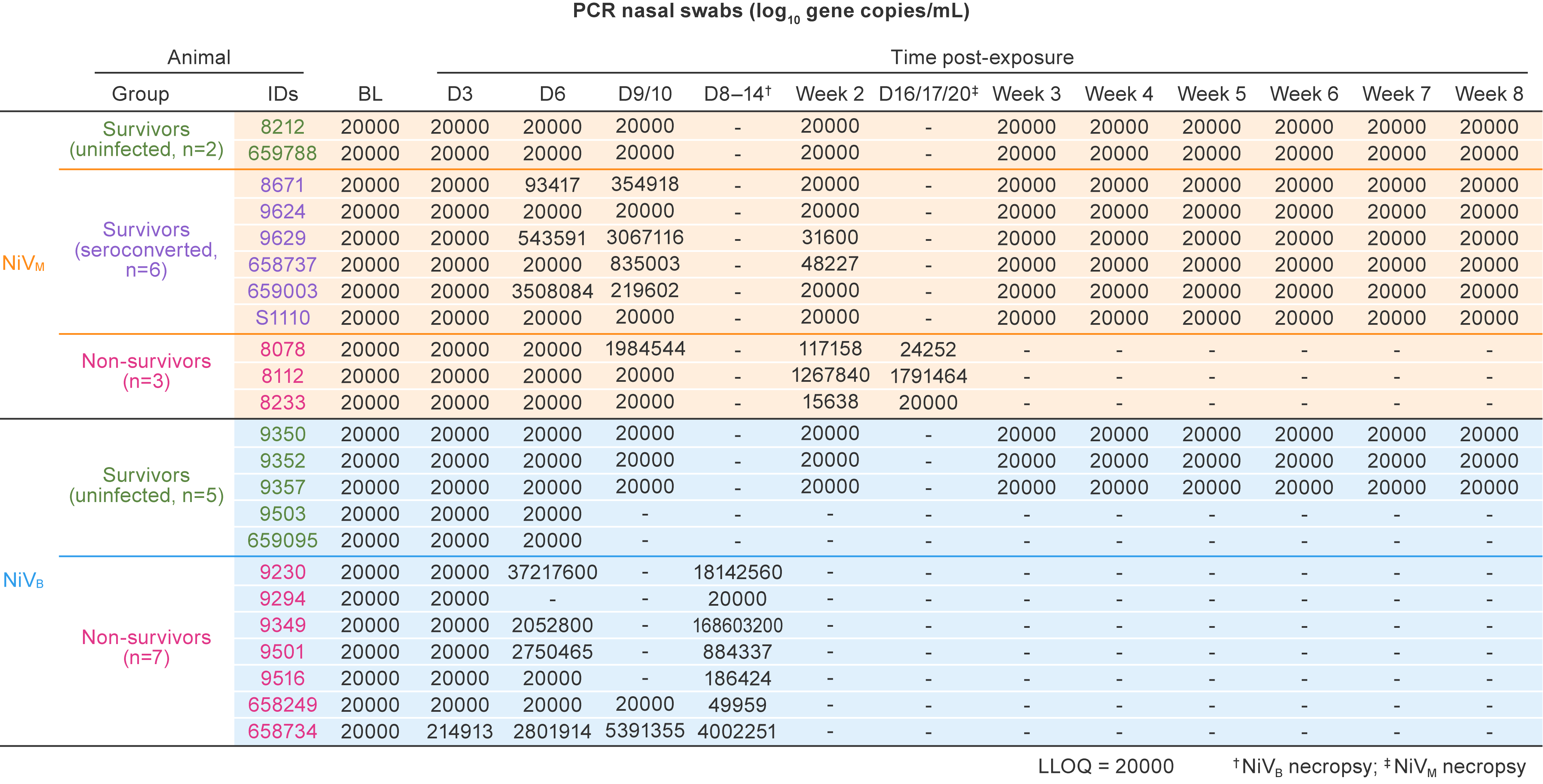

Supplement: S19 Fig — (TIF) [file ppat.1013835.s019.tif]

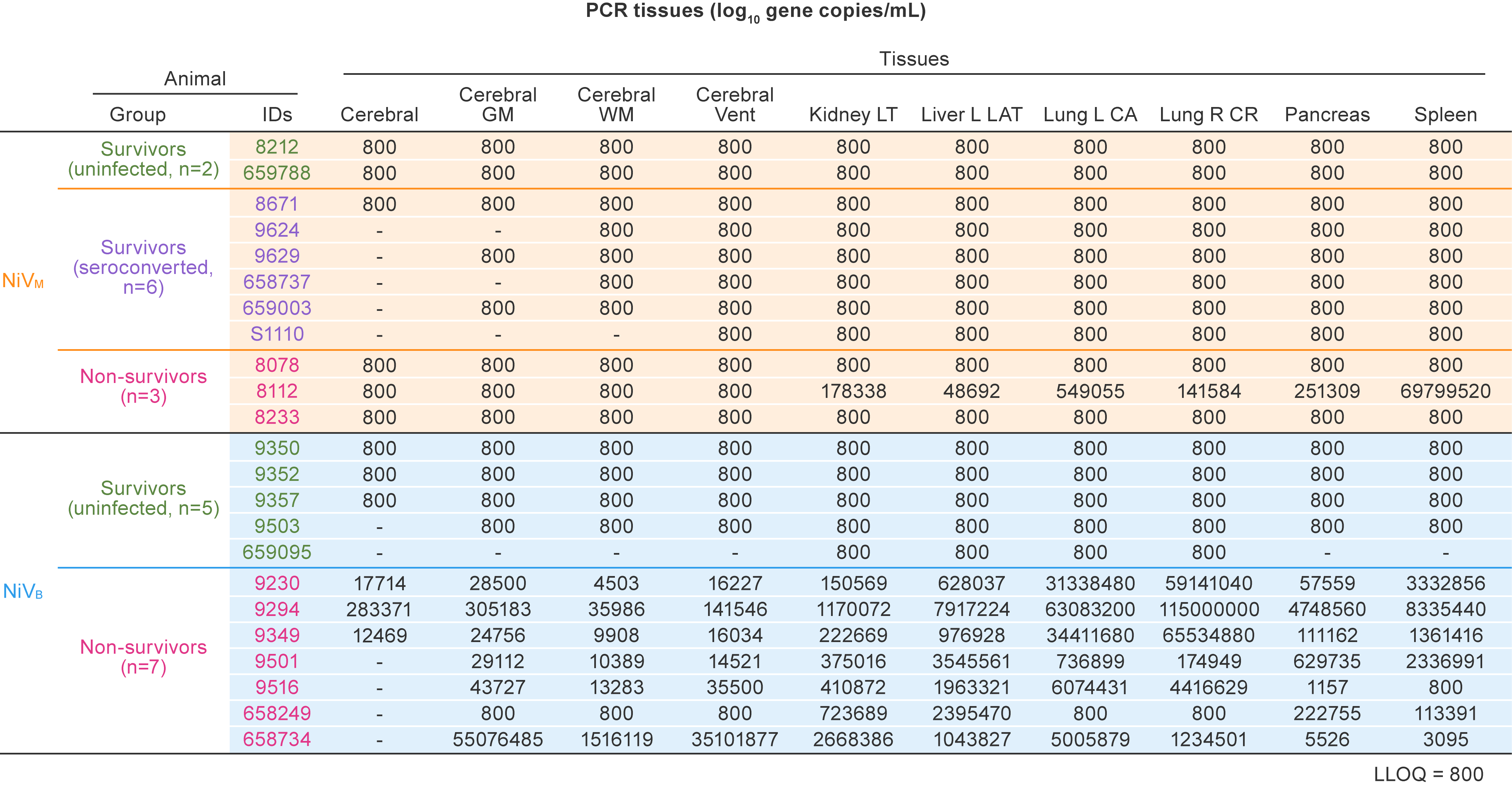

Supplement: S20 Fig — (TIF) [file ppat.1013835.s020.tif]

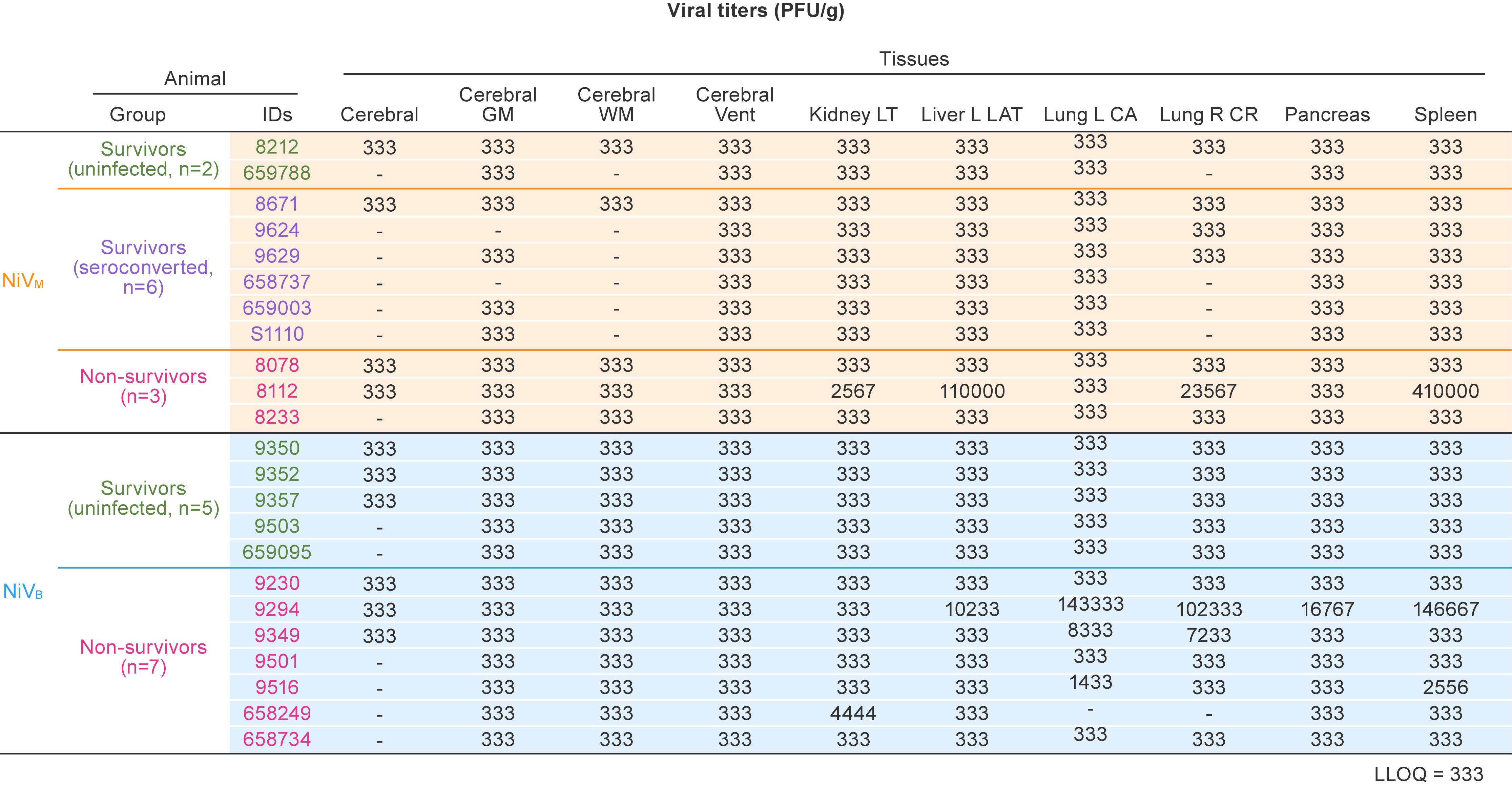

Supplement: S21 Fig — (TIF) [file ppat.1013835.s021.tif]

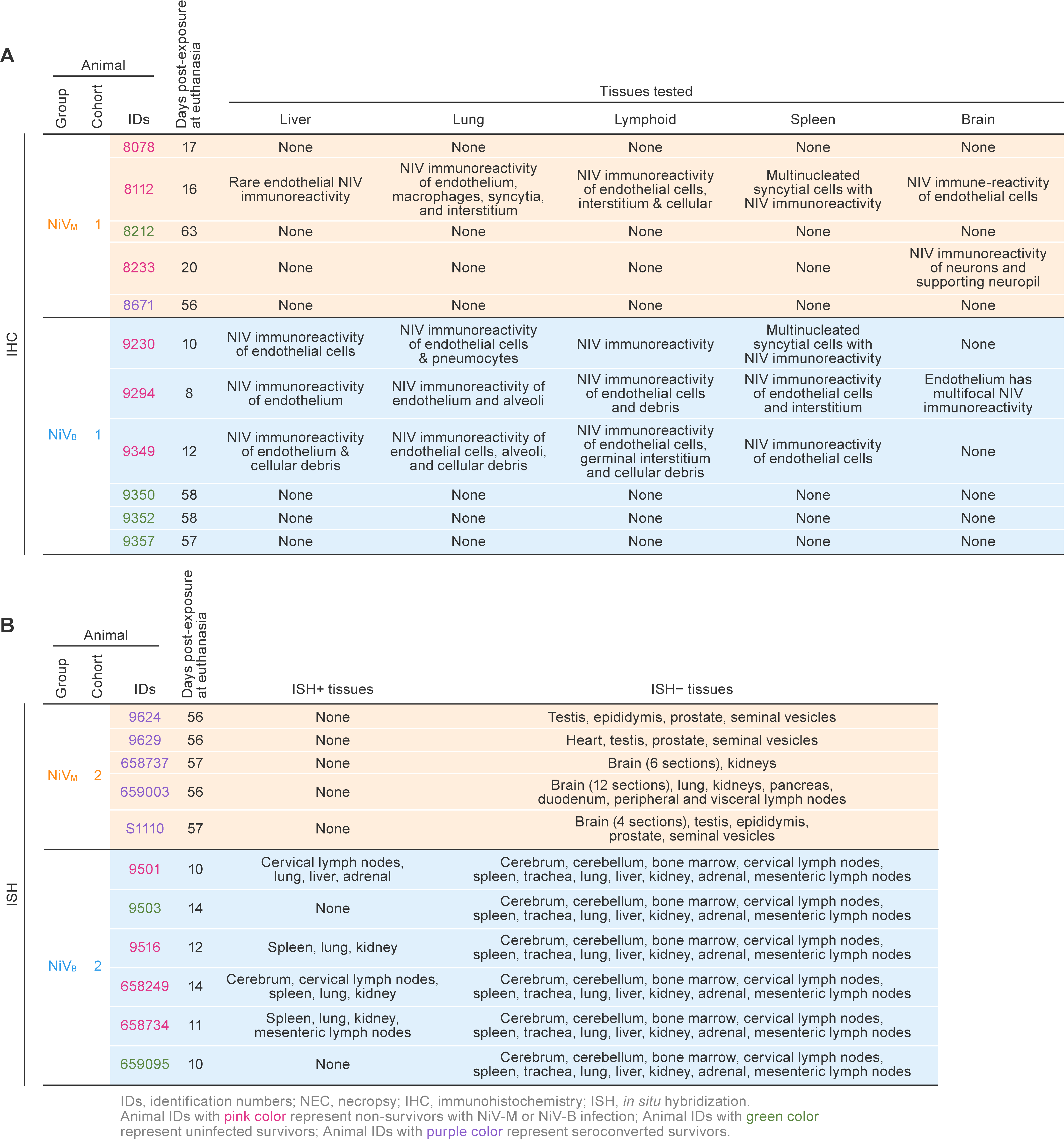

Supplement: S22 Fig — (TIF) [file ppat.1013835.s022.tif]
